# Complete field-induced spectral response of the spin-1/2 triangular-lattice antiferromagnet CsYbSe_2_

**DOI:** 10.1038/s41535-023-00580-9

**Published:** 2023-09-23

**Authors:** Tao Xie, A. A. Eberharter, Jie Xing, S. Nishimoto, M. Brando, P. Khanenko, J. Sichelschmidt, A. A. Turrini, D. G. Mazzone, P. G. Naumov, L. D. Sanjeewa, N. Harrison, Athena S. Sefat, B. Normand, A. M. Läuchli, A. Podlesnyak, S. E. Nikitin

**Affiliations:** 1 Neutron Scattering Division, Oak Ridge National Laboratory, Oak Ridge, TN 37831 USA; 2 Institut für Theoretische Physik, Universität Innsbruck, Innsbruck, Austria; 3 Materials Science and Technology Division, Oak Ridge National Laboratory, Oak Ridge, TN 37831 USA; 4 Department of Physics, Technical University Dresden, 01069 Dresden, Germany; 5 Institute for Theoretical Solid State Physics, IFW Dresden, 01069 Dresden, Germany; 6 Max Planck Institute for Chemical Physics of Solids, Nöthnitzer Str. 40, D-01187 Dresden, Germany; 7 Laboratory for Neutron Scattering and Imaging, Paul Scherrer Institut, CH-5232 Villigen-PSI, Switzerland; 8 Quantum Criticality and Dynamics Group, Paul Scherrer Institut, CH-5232 Villigen-PSI, Switzerland; 9 Orange Quantum Systems B.V., Elektronicaweg 2, 2628 XG Delft, The Netherlands; 10 National High Magnetic Field Laboratory, Los Alamos National Laboratory, Los Alamos, NM 87545 USA; 11 Laboratory for Theoretical and Computational Physics, Paul Scherrer Institut, CH-5232 Villigen-PSI, Switzerland; 12 Institute of Physics, Ecole Polytechnique Fédérale de Lausanne (EPFL), CH-1015 Lausanne, Switzerland

**Keywords:** Magnetic properties and materials, Magnetic properties and materials

## Abstract

Fifty years after Anderson’s resonating valence-bond proposal, the spin-1/2 triangular-lattice Heisenberg antiferromagnet (TLHAF) remains the ultimate platform to explore highly entangled quantum spin states in proximity to magnetic order. Yb-based delafossites are ideal candidate TLHAF materials, which allow experimental access to the full range of applied in-plane magnetic fields. We perform a systematic neutron scattering study of CsYbSe_2_, first proving the Heisenberg character of the interactions and quantifying the second-neighbor coupling. We then measure the complex evolution of the excitation spectrum, finding extensive continuum features near the 120°-ordered state, throughout the 1/3-magnetization plateau and beyond this up to saturation. We perform cylinder matrix-product-state (MPS) calculations to obtain an unbiased numerical benchmark for the TLHAF and spectacular agreement with the experimental spectra. The measured and calculated longitudinal spectral functions reflect the role of multi-magnon bound and scattering states. These results provide valuable insight into unconventional field-induced spin excitations in frustrated quantum materials.

## Introduction

Frustrated quantum magnets provide an intriguing playground for investigating novel many-body phenomena in condensed matter^1,2^. The triangular-lattice Heisenberg antiferromagnet (TLHAF) is a prototypical example of geometrical frustration, and its ground state in the quantum limit of *S* = 1/2 spins has the 120° AF order of the classical (large-*S*) case^3^, albeit with an ordered moment strongly suppressed by quantum fluctuation effects (Ref. ^4^ and references therein). Proposals to capture these effects include the resonating valence-bond (RVB) paradigm^5^, and the addition of a weak next-neighbor HAF interaction (0.06 ≲ *J*_2_/*J*_1_ ≲ 0.15) does drive the *S* = 1/2 TLHAF into a quantum spin-liquid (QSL) phase^6–9^ of some type^10–12^. Over a finite range of applied magnetic fields, AF quantum fluctuations favor a collinear up-up-down (UUD) ordered phase and thus stabilize a magnetization plateau with *M* = *M*_Sat_/3 (where *M*_Sat_ is the saturation magnetization)^13,14^.

Theoretical research on the TLHAF has been driven by new generations of TL materials. The Cs_2_Cu*X*_4_ compounds (*X* = Cl, Br)^15,16^ inspired studies of spatially anisotropic TLs^3^. The low-spin cobaltates Ba_3_Co*X*_2_O_9_ (*X* = Nb, Sb)^17–21^ and Ba_8_CoNb_6_O_24_^22^ focused attention on XXZ spin anisotropy^14^. The first 4*f* TLAF, YbMgGaO_4_^23,24^, sparked more extensive studies of spin anisotropy that enriched the phase diagram and revealed the connection to the QSL phase of the *J*_1_-*J*_2_ TLHAF^25–27^.

**Fig. 1:**
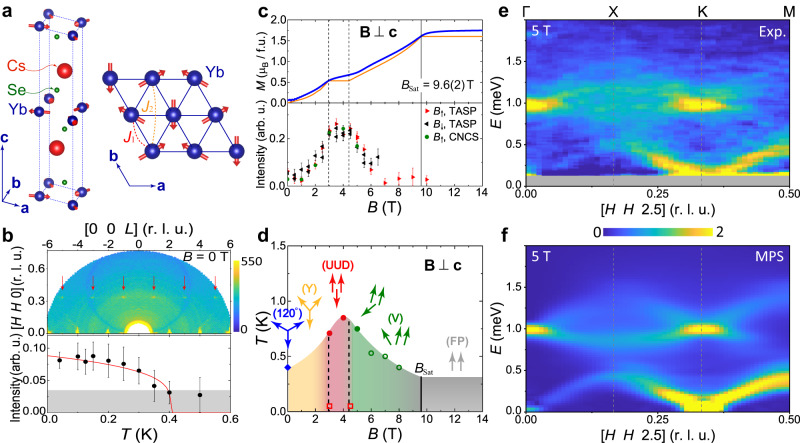
Structure, properties, phase diagram and finite-field spectra of CsYbSe_2_. **a** Crystal structure of CsYbSe_2_ and representation of the ideal Yb^3+^ TL layer. The red arrows represent the ordered spins of the weak 120° AF order at zero field. **b** Upper panel: elastic scattering intensity in the (*HHL*) plane for 0 T, with the data presented as described in Supplementary Note 2B. Red arrows indicate weak magnetic intensity peaks at **Q** = (1/3, 1/3, ± *L*) with *L* = 1, 3, 5. Lower panel: temperature-dependence of the (1/3, 1/3, 1) peak area at zero field. The red solid line is a fit to an order-parameter form. The gray shaded area represents the approximate sensitivity limit of our measurement. **c** Upper panel: isothermal magnetization (blue symbols) measured at *T* = 0.4 K as a function of magnetic field applied in the *ab* plane and with the van Vleck contribution subtracted as described in Supplementary Note 1C. The solid orange line shows a grand canonical density-matrix renormalization-group (DMRG) calculation of the magnetization performed for the TLHAF using parameters deduced in Fig. 2, from which we determined the saturation field, *B*_Sat_ = 9.6(2) T (vertical solid line), and the lower and upper boundaries of the 1/3 plateau as *B*_l_ = 2.95(6) T and *B*_u_ = 4.5(1) T (vertical dashed lines). Lower panel: integrated intensity of the (1/3, 1/3, 1) magnetic Bragg peak measured at *T* < 0.05 K. **d** Phase diagram of CsYbSe_2_. The blue diamond is the phase-transition temperature obtained from neutron diffraction at zero field. The two open squares indicate the region (3 T ≤ *B* ≤ 4.5 T) where the peak intensity in the lower half of **c** remains almost unchanged. The solid and open circles represent respectively the temperatures of sharp peaks and broad humps in the corresponding specific-heat curves, as described in Supplementary Note 1D. The arrows indicate schematically the spin order of the five phases of the classical TLHAF, which are consistent with the magnetic peaks we observe. **e** INS spectrum measured under a magnetic field of 5 T, showing an absence of well defined Δ*S* = 1 excitations away from the Γ and K points but extensive continuum features (Fig. 3). The gray horizontal bar at low energy masks the elastic-line contribution. **f** Comparison with a matrix-product-state (MPS) calculation of the spectrum at the same field.

In this context, the Yb-based delafossite family *A*Yb*Q*_2_, with *A* an alkali metal and *Q* a chalcogenide, has attracted widespread attention^28,29^. The Yb ions form perfect and well separated TLs (Fig. 1a)^30–32^ without the structural disorder intrinsic to YbMgGaO_4_^33,34^. The combination of strong spin-orbit coupling and the crystalline electric field (CEF) creates a ground-state doublet that gives an effective *S* = 1/2 pseudospin at low temperatures^30,31,35,36^. Although the *J* = 7/2 CEF level structure is manifest in a strong spatial anisotropy of the response to applied magnetic fields^37,38^, initial scattering studies provided no evidence for a strongly non-Heisenberg pseudospin Hamiltonian^39,40^. Early specific heat, magnetization, muon spin-rotation spectroscopy and neutron diffraction studies of multiple *A*Yb*Q*_2_ materials found no magnetic order at zero field down to their base temperatures^31,37,39–41^, but recent studies, including our own (Fig. 1b), indicate its presence in some materials at the 0.1 K scale. The 1/3-magnetization plateau is found at in-plane fields in the 3–5 T range^37,39,40,42^ (Fig. 1c), with robust UUD order up to 1 K. Clearly, the delafossite family offers an excellent platform to study the field-controlled magnetic states of the *S* = 1/2 TLAF.

Initial inelastic neutron scattering (INS) measurements on single-crystalline Yb delafossites at zero field^40,43^ suggested a gapless excitation continuum, which was interpreted as originating from a QSL ground state, but appears to persist even in the presence of weak magnetic order^44^. Early INS studies of the spin dynamics in the field-induced phases were limited by their polycrystalline samples^39,45^, but the 1/3-magnetization plateau has recently been analyzed in some detail^46^. As in Ba_3_CoSb_2_O_9_, where the plateau has been reached despite the higher energy scales in this family of materials^47^, the magnetic excitations were captured largely by semiclassical nonlinear spin-wave theory (SWT). The lower in-plane energy scale and vanishing inter-plane coupling in delafossites present more experimental challenges, but also the key advantage of reaching saturation within laboratory-available magnetic fields.

On the theoretical side, the challenge of computing the dynamical spectral functions of frustrated models lies in the absence of analytical methods that capture all the physics of non-collinear magnetic states with a field-controlled ratio of weak order to strong quantum fluctuations. The application of unbiased numerical methods, meaning those whose truncation methodology can be extended systematically to convergence, has in the past been impossible, but continuous progress in dynamical quantum Monte Carlo techniques and matrix-product-state (MPS) representations is placing this goal within reach. For the TLHAF, the zero-field spectral function has been obtained by a number of biased methods, by which we mean those based on initial assumptions that have to be assessed *a posteriori*; these include series expansions^48^, interacting spin waves^49,50^, Schwinger bosons^51,52^, bond operators^53^ and variational Monte Carlo^54^. Despite recent progress with MPS calculations in a cylinder geometry^11,12^, the full field-induced dynamics has remained an unsolved problem. All of these methods produce scattering continua whose origin may lie in fractional excitations, multi-magnon states, or possibly neither.

In this work, we perform high-resolution neutron spectroscopy on CsYbSe_2_ using two different spectrometers to span the full range of applied in-plane fields, meaning from zero to beyond saturation. Our measurements reveal the pronounced changes in the magnetic excitation spectrum as it evolves with the magnetic field, and we associate these with the field-driven phase transitions of the ground state (Fig. 1d). In parallel, we perform large-cylinder MPS calculations of the full TLHAF spectral function at all fields to obtain a hitherto unavailable benchmark for the model, semi-quantitative agreement with experiment (Fig. 1e–f) and a robust foundation for any effective quasiparticle descriptions of the spin dynamics.

## Results

### Ground state at zero field

The growth and structural characterization of our single crystals are summarized in the Methods section and detailed in Supplementary Note 1A. Neutron diffraction at zero magnetic field (Supplementary Note 2) reveals a series of weak magnetic intensity peaks at **Q** = (1/3, 1/3, *L*) for odd-integer *L* (Fig. 1b). The (1/3, 1/3, 1) peak develops a finite intensity below *T* ≃ 0.4 K, which increases on cooling. The propagation wavevector matches the 120° state of the TLHAF with AF out-of-plane correlations (represented by the arrows in Fig. 1a) and the low-temperature ordered moment is *m*_Yb_ ≃ 0.1*μ*_B_. In Supplementary Note 2B, we extract the in- and out-of-plane correlation lengths, *ξ*_*ab*_ = 60(7) Å and *ξ*_*c*_ = 23(5) Å, which are not resolution-limited, meaning that CsYbSe_2_ does not exhibit true, long-ranged AF order at zero field down to *T* = 0.02 K. However, the presence of the magnetic peak clearly excludes a QSL, as in KYbSe_2_^44^ but in contrast to NaYbSe_2_^43^. Given that CsYbSe_2_ (space group P6_3_/*mmc*) has AA layer stacking (Fig. 1a), which should favor an unfrustrated collinear *c*-axis order, whereas the Na, K, and Rb materials (space group R$\overline{3}m$) have an ABC stacking that should produce interlayer frustration, we suggest in Supplementary Note 1B that the origin of this behavior may instead lie in the next-nearest neighbor coupling, *J*_2_ (below).

### Magnetic phase diagram of CsYbSe_2_

**Fig. 2:**
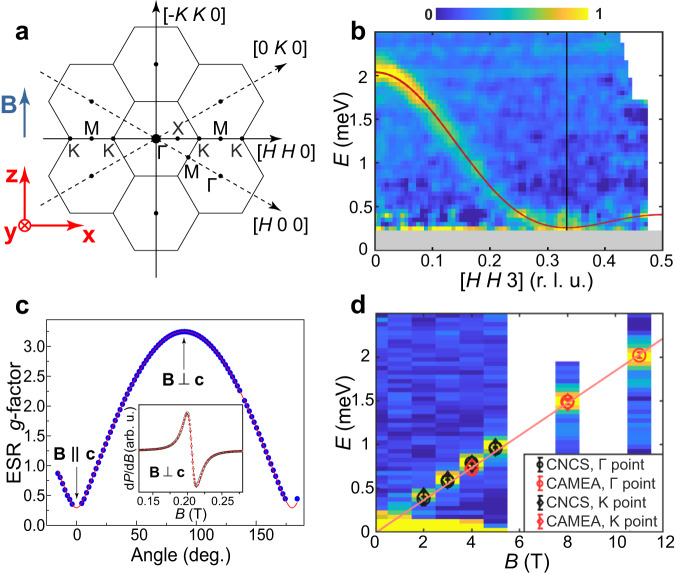
INS and ESR determination of the spin Hamiltonian. **a** Representation of the Brillouin zone in the (*HK*0) plane and definition of directions $\hat{x}\parallel \,[1\,1\,0]$, $\hat{y}\parallel \,[0\,0\,1]$ and $\hat{z}\parallel {{{\bf{B}}}}$, which is orthogonal to $\hat{x}$ and $\hat{y}$. **b** Spin excitations along the [*HH*3] direction measured on CAMEA at *T* = 0.05 K in the field-polarized regime (*B* = 11 T). The solid line shows the dispersion calculated using linear spin-wave theory (SWT) with *J*_1_ = 0.395(8) meV, *J*_2_ = 0.011(4) meV and *g* = 3.2. The gray horizontal bar at low energy masks the elastic-line contribution. **c** Angular dependence of the *g*-factor measured by ESR at *T* = 15 K. The solid line indicates the form $g(\theta )=\sqrt{{g}_{ab}^{2}{\sin }^{2}\theta +{g}_{c}^{2}{\cos }^{2}\theta }$, which allows the extraction of the strongly anisotropic in- and out-of-plane coefficients *g*_*ab*_ = 3.25 and *g*_*c*_ = 0.3. Inset: representative ESR spectrum with a Lorentzian fit shown by the dashed red line. **d** Field-dependence of the INS signal at the Γ point; data for *B* ≤ 5 T were measured on CNCS and data for *B* = 8 and 11 T on CAMEA. Black and red points show the respective positions of the magnon mode as extracted from the CNCS and CAMEA datasets, for both the Γ and K points. The solid line shows a linear fit that yields *g*_*ab*_ = 3.20(6).

We performed low-temperature magnetization, specific-heat and neutron diffraction measurements over a wide field range, as described in Supplementary Notes 1 and 2. Figure 1c shows isothermal magnetization data, with evidence of a plateau at *M*_Sat_/3 corresponding to the UUD phase^13,14^. To interpret these data despite their finite-temperature rounding, we estimate *B*_Sat_ = 9.6(2) T from the TLHAF model parameters obtained by high-field INS (Fig. 2) and perform a grand canonical DMRG calculation^55^ of the magnetization (Supplementary Note 4) that allows us to deduce the boundaries of the 1/3-magnetization plateau. We compare these data with the integrated intensity of the (1/3, 1/3, 1) magnetic peak, which increases strongly from 0 T to the UUD state, then remains maximal and almost constant over the field range of the plateau (3 T ≤ *B* ≤ 4.5 T), before decreasing strongly towards the fully polarized (FP) state. Our thermodynamic and neutron diffraction results yield the field-temperature phase diagram shown in Fig. 1d, where we indicate the spin alignments of the classical TLHAF.

### Parameters of the magnetic Hamiltonian

We made INS measurements up to 5 T on the time-of-flight (ToF) spectrometer CNCS at ORNL and up to 11 T on the multiplexing spectrometer CAMEA at PSI, as detailed in the Methods section. To quantify the parameters of the spin Hamiltonian, we exploit our ability to perform INS at fields *B* > *B*_Sat_, where the magnetic excitations of the FP phase can be described by linear SWT. Figure 2b shows that the spectrum measured along [*HH*3] at 11 T consists of a single, sharp magnon mode with a cosinusoidal dispersion above a field-induced gap at the K point. In Supplementary Note 2D, we show CNCS data indicating a complete lack of out-of-plane dispersion over the whole field range, and hence that a two-dimensional TL model is appropriate. We fit this dispersion using the SPINW package^56^ by considering a Hamiltonian with an anisotropic XXZ-type *J*_1_ term and a Heisenberg *J*_2_ term (Supplementary Note 3A). With the in-plane *g*-factor fixed (below), the optimal fit (solid red line in Fig. 2b) yields two essential pieces of information. First, the nearest-neighbor interaction has no XXZ anisotropy within the precision of the measurement, i.e., despite the strongly anisotropic field response^29,38^, the spin dynamics are of Heisenberg type; in ref. ^35^ it was shown how these contrasting forms of behavior can appear simultaneously in edge-sharing octahedral Yb^3+^ systems. Second, the next-neighbor interaction is sufficiently weak, *J*_2_/*J*_1_ ≃ 0.03, that CsYbSe_2_ remains on the ordered side of the phase boundary separating the 120^∘^ and QSL states in the *S* = 1/2*J*_1_–*J*_2_ TLHAF^6–9^. We therefore conclude that a *J*_1_–*J*_2_ Heisenberg Hamiltonian

1 $${{{\mathcal{H}}}}={J}_{1}\mathop{\sum}\limits_{\langle i,j\rangle }{{{{\bf{S}}}}}_{i}\cdot {{{{\bf{S}}}}}_{j}+{J}_{2}\mathop{\sum}\limits_{\langle \langle i,j\rangle \rangle }{{{{\bf{S}}}}}_{i}\cdot {{{{\bf{S}}}}}_{j}-{\mu }_{B}{g}_{ab}B\mathop{\sum}\limits_{i}{S}_{i}^{z},$$

with *J*_1_ = 0.395 meV, provides a complete description of the low-energy magnetic behavior in CsYbSe_2_.

Turning to fields below saturation, we begin in Fig. 2d by considering constant-**Q** cuts at the Γ-point for each field. These show a clear, single-peak feature for *B* ≥ 2 T, whose energy obeys the field-linear form ℏ*ω*(*B*) = ℏ*ω*_0_ + *g*_*ab*_*μ*_B_*BS* with *g*_*ab*_ = 3.20(6) and ℏ*ω*_0_ = 0.00(2) meV. This field-induced behavior at Γ is generic for the Heisenberg model^57^, reinforcing our conclusion concerning the absence of XXZ anisotropy, and in the TLHAF is also present at K (Fig. 2d). To characterize the anisotropic field response, we have performed electron spin resonance (ESR) measurements that determine the *g*-tensor parameters shown in Fig. 2c. The narrow and well-defined ESR spectrum reflects the high quality of our crystal. The best fit in Fig. 2c, *g*_*ab*_ = 3.25(0) and *g*_*c*_ = 0.3(0), completes our determination of the Hamiltonian parameters for CsYbSe_2_ in any applied field and also shows the consistency of our INS result for *g*_*ab*_. Our measurements also demonstrate that *g*_*ab*_ is isotropic in the *ab* plane to within the experimental accuracy (data not shown). The very small *g*_*c*_ is a consequence of strong hybridization with the first excited CEF doublet^38^, and we comment below on its role in our scattering study.

### MPS calculations of spectral functions

**Fig. 3:**
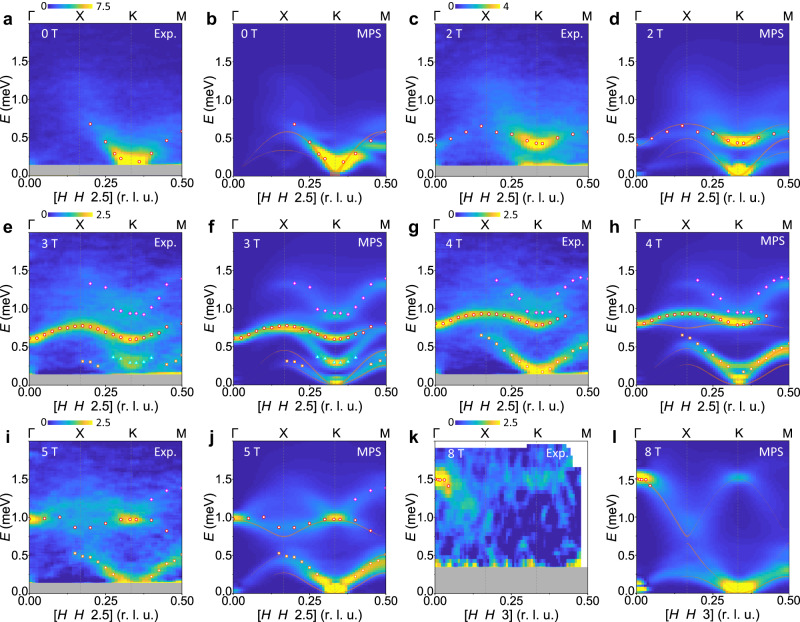
Complete field-induced spectral response of CsYbSe_2_. **a**, **c**, **e**, **g**, **i,** and **k** show spin excitation spectra measured under different magnetic fields at *T* = 0.07 K. The open circles, squares, triangles, and diamonds indicate respectively excitation features in the categories I, II, III, and IV described in the text. All data have been symmetrized according to the crystal symmetry. The orthogonal in-plane integration range along the [−*KK*0] direction is *K* = [−0.05, 0.05] and the out-of-plane range is 1.2 ≤ *L* ≤ 3.8 for our CNCS data (0–5 T) and 2 ≤ *L* ≤ 4 on CAMEA (8 T). The background subtraction is described in Supplementary Note 2E. The narrow horizontal gray regions mask the elastic line. **b**, **d**, **f**, **h**, **j,** and **l** present dynamical spin structure factors obtained at different magnetic fields by cylinder MPS calculations (Supplementary Note 5). Color bars represent both the measured and calculated intensities in a single set of arbitrary units (i.e., the same units are used at all fields). Orange lines show the mode positions and intensities given by linear SWT with the same interaction parameters. The open points are identical to those shown in **a**, **c**, **e**, **g**, **i,** and **k**.

To interpret the measured spin dynamics, we have performed MPS calculations on a finite cylinder to obtain the dynamical spectral function of the TLHAF with *J*_2_ = 0.03*J*_1_, where the energy unit is fixed to *J*_1_ = 0.395 meV. The cylinder size, matrix bond dimensions and time-evolution procedures are summarized in the Methods section and their convergence to the properties of the TLHAF is benchmarked in Supplementary Note 5. We compute the spin correlation functions both transverse and longitudinal to the applied field, which in the notation of Fig. 2a are respectively *S*_*xx*_(**Q**, *ω*) and *S*_*zz*_(**Q**, *ω*). In experiment, the strong *g*-tensor anisotropy (*g*_*ab*_ ≫ *g*_*c*_) means that the component fluctuating parallel to the *c* axis (*S*_*yy*_) is hidden [Fig. 2a defines the (*HKL*) and (*xyz*) coordinate frames]. The measured intensities then represent the sum of *S*_*xx*_ and *S*_*zz*_ weighted by the polarization factor, which ensures that spectra taken along [*HH*0] have no contribution from *S*_*xx*_, i.e., only from the component longitudinal to the applied field. In order to sample both components, in Fig. 3, we integrate our INS and MPS spectra over a wide range of the out-of-plane momentum, *L*.

### Field-induced evolution of the spectrum

As expected from the phase diagram (Fig. 1d), both the observed and computed spectra in Fig. 3 are readily classified by their field-induced evolution into four regimes, to which we refer as Y, UUD, V and FP (the last analysed in Fig. 2). Starting with Y, we have shown (Fig. 1b) that the zero-field ground state of CsYbSe_2_ is consistent with three-sublattice 120° order, and thus the spectrum should contain three excitation branches. However, both the INS and MPS spectra exhibit only a broad, V-shaped continuum around K (Fig. 3a, b), similar to the spectra observed in NaYbSe_2_^43^ and KYbSe_2_^44^. Because this clear signature of strong quantum fluctuations on top of weak magnetic order has received extensive theoretical^49,51,58^ and numerical analysis^11,12,44,48,54^, which our results confirm but do not extend, we focus rather on adding to the understanding of the finite-field spectra.

At 2 T (Fig. 3c, d), most of the spectral intensity shifts upwards, forming the broad feature, with a gap around 0.4 meV at Γ and K, seen in Fig. 2d. Away from Γ and K, however, it has no well defined magnonic form and the spectrum appears as a weak and highly dispersed continuum. Nevertheless, we mark the maximum intensity of this feature by the circles in Fig. 3c, d and refer to it as mode I, observing its bandwidth falling rapidly across the Y regime. Another mode is present at lower energies, whose gapless nature is clearly visible in the MPS spectrum. Linear SWT captures rather well the maximum of mode I, and also finds two gapless branches, but cannot reproduce the extreme broadening of the measured modes away from Γ and K, their intensity distribution or the continuum scattering above mode I.

**Fig. 4:**
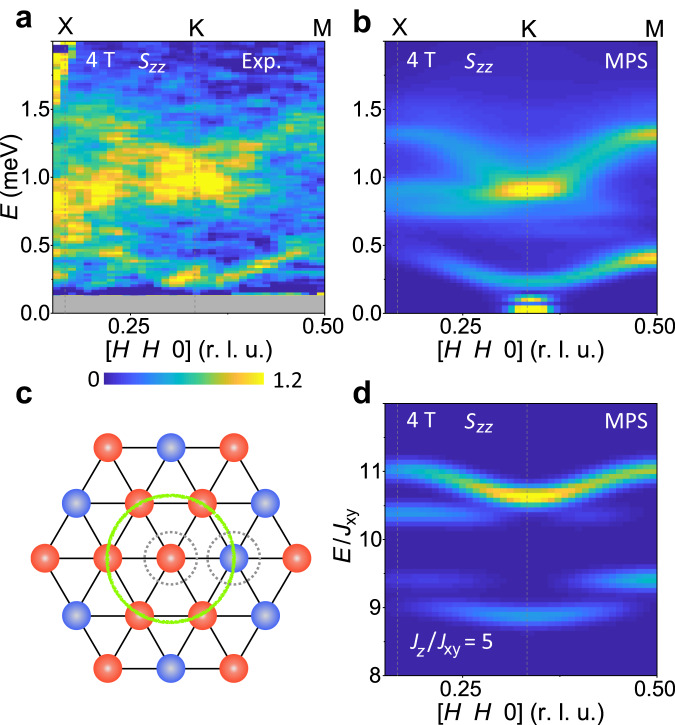
Longitudinal spin excitations. **a** Longitudinal component of the excitation spectrum extracted for the [*HH*0] direction at *B* = 4 T. The orthogonal in-plane integration range is *K* = [−0.05, 0.05] and the out-of-plane range is *L* = [ − 0.5, 0.5]. The narrow gray region masks the elastic line. **b** Corresponding MPS calculation of the longitudinal component, *S*_*zz*_(**Q**, *ω*), of the dynamical structure factor. **c** Schematic representation of spin-flip processes in the UUD phase: red and blue circles represent respectively U and D spins, the gray dashes highlight flipped spins (U → D and D → U) and the green circle delineates the hexagon on which the blue flipped spin may propagate at no energy cost in the Ising limit, ensuring its localization. **d** MPS calculation of *S*_*zz*_(**Q**, *ω*) for the strongly Ising-type parameter choice *J*_*z*_ = 5*J*_*xy*_ (Supplementary Note 7); the mode energies are shown in units of *J*_*xy*_ to illustrate the role of this interaction in setting the splitting and dispersion of the bound states centered at 2*J*_1_ = 2*J*_*z*_.

The data collected at 3 and 4 T represent, respectively, the lower edge and upper middle of the UUD regime (Fig. 1c). In contrast to the Y phase, a number of rather sharp excitations extend across the full Brillouin zone, and at 3 T we identify four distinct features (Fig. 3e, f). Mode I shifts upwards, becomes resolution-limited and has intensity over a large **Q** range. A weak and very low-lying feature II is visible only around its maximum near 0.4 meV. A broad feature III is concentrated around the K point and disperses upwards to touch mode I. A continuum feature IV disperses from around 0.8 meV at K to 1.3 meV at X and M. At 4 T (Fig. 3g, h), features II and III have almost merged to become a strong, spin-wave-like branch. Mode I continues its upward shift while continuum IV remains almost unchanged in position and intensity. Modes I-III have been observed in Ba_3_CoSb_2_O_9_^47^, and very recently, all four features were measured in KYbSe_2_^46^. Both studies used a nonlinear SWT to obtain a good account of modes I-III, and in KYbSe_2_ it was suggested that feature IV is a two-magnon continuum. Given that the 1/3 plateau is absent in linear SWT, it is not surprising that the orange lines in Fig. 3f and h provide at best partial agreement with some observed branches. By contrast, our MPS calculations provide a quantitatively excellent description of every feature in the observed UUD spectra, which will allow a deeper analysis in Fig. 4.

Proceeding into the V phase at 5 T causes a qualitative modification of the spectrum (Fig. 3i, j). As in the Y phase, no clear magnon branches are visible away from Γ and K. More specifically, mode II softens, broadens, and decreases in intensity, while mode III merges fully with it. Mode I decays into a broad continuum with sharp intensity peaks only at Γ and K, and obscures continuum IV. Linear SWT traces only the lower boundary of the mode-I continuum and the dispersion of mode II. The 8 T dataset in Fig. 3k shows a sharp band maximum at Γ (mode I) together with a weak replica at K. Here our MPS results (Fig. 3l) clarify how mode I becomes very broad and mode II becomes very soft; linear SWT provides an acceptable guide to the positions, but absolutely not to the emerging mid-zone continuum nature, of these features. In Supplementary Note 6, we present cuts through the data displayed in Fig. 3a–j that confirm the near-quantitative agreement between the INS and MPS spectra at almost all points in **Q** and *ω*.

### Two-magnon bound and scattering states

To analyse the spectra in Fig. 3, we begin in the UUD phase, where all the ordered moments are orientated (anti)parallel to the field and thus the *S*_*zz*_ channel contains purely those spin fluctuations longitudinal to the field. Figure 4a shows the longitudinal excitation spectrum obtained from a data slice in the [*HH*0] direction and Fig. 4b shows the analogous MPS calculation. Both spectra show a weakly dispersive, low-energy branch running from X to M, and above this the entirety of continuum IV. To understand the origin of these longitudinal features, we appeal first to the Ising limit, where in the UUD phase a single spin-flip against the field direction costs no energy, whereas the opposite flip costs 3*J*_1_. If both processes occur on neighboring spins, the energy cost is only 2*J*_1_ (Fig. 4c), forming a localized two-magnon bound state. The spectrum close to the Ising limit then contains a nearly flat bound-state mode at an energy of 2*J*_1_, which is clearly split off from a continuum of states that starts around 3*J*_1_ (Supplementary Note 7). In Fig. 4d, we show an MPS calculation at rather strong Ising anisotropy that nevertheless shows many properties of the Heisenberg case (Fig. 4b), and in Supplementary Fig. 16, we show a more complete interpolation. These results demonstrate clearly the evolution of the lowest localized modes into a split-off and weakly dispersive longitudinal two-magnon bound state, while the upper localized modes evolve into a scattering resonance that forms the characteristic shape of continuum IV. Thus the Ising picture of these features remains valid even at the Heisenberg point.

Increasing the field into the V phase (Fig. 3i, j) causes the longitudinal spectrum to show little change, whereas the transverse magnons disintegrate rapidly. Increasing non-collinearity leads to a mixing of transverse and longitudinal character, such that both sets of excitations merge into narrow continua (on the scale of the bandwidth) with strong intensity concentrated only at the Γ and K points. These continuum features become both sharper and more dispersive with increasing field, regaining their single-magnon character above *B*_Sat_ (Fig. 2b). By contrast, as the field is decreased into the Y phase (Fig. 3c, d), the effects of non-collinearity and dominant quantum fluctuations lead to a rapid loss of one-magnon character (again intensity is concentrated only at the Γ and K points) and the emergence of wide excitation continua in both *S*_*zz*_ and *S*_*xx*_.

## Discussion

Our INS measurements demonstrate unambiguously that the excitations of the TLHAF at all fields consist of magnon-like features only around the Γ and K points that merge into extensive continua across the rest of the Brillouin zone. Despite the presence of at least short-ranged magnetic order at all fields, only in the UUD (1/3-plateau) phase can single magnons provide an adequate basis for describing the spectrum. Capturing the effects of strong quantum fluctuations on such weak order remains a major challenge, which we address by cylinder MPS calculations of the spectrum. Deploying such an unbiased numerical method allows one to divide the process of obtaining physical understanding into a two-step exercise of “expression” and “interpretation,” but brings into focus a dichotomy between the two. The expressibility of the MPS method is excellent, in that it captures all the features of the measured excitations with semi-quantitative accuracy, but as a numerical experiment, its interpretability is limited. The primary contribution of our study is at the first step, namely providing an unbiased approach that confirms the true spectral content of a paradigm model. At least for the TLHAF, several different biased methods exist that interpret some of the observed spectral features, but to date have lacked a benchmark. Here it is the agreement between our INS and MPS results, which allows us to assert that we have delivered the required benchmark.

We have, in addition, provided a modern standard for theoretical methods by employing the applied field as a control parameter to access four different, but continuously connected, physical regimes. Thus the ability to separate the transverse and longitudinal spectral functions in one regime affords some key insight that we use to interpret the longitudinal response in the other regimes. When we do consider one biased approach to interpreting the measured and calculated spectra, we find the hallmarks of bound and resonant states of magnon pairs. In the literature it has been argued that scattering continua can arise either from fractionalization (into bosonic^51,52,58^ or fermionic^54^ components) or from the formation of two-particle and higher-order bound and scattering states of spin-1 excitations^48,49,59^, a subset of the latter being the magnon-breakdown scenario^60,61^. Although none of our present results necessitate a fractionalization scenario to explain the observed spectra, we certainly cannot exclude that fully quantitative analyses of the low-field limit could yet reveal the presence of deconfining *S* = 1/2 entities in the TLHAF.

To place our results in perspective, to our knowledge, the complete field-induced spectrum of a 2D Heisenberg system has not previously been determined in experiment, and here we provide it for the TLHAF realized in CsYbSe_2_. Methodologically, we have used our spectral data to benchmark cylinder MPS calculations of the dynamical spectral function at all applied fields, demonstrating that these now provide a powerful numerical method delivering near-quantitative accuracy. The next-neighbor TLHAF with *J*_2_ on the cusp of the QSL phase provides a microcosm of all the key questions in quantum magnetism, arising where strong quantum spin fluctuations cause a partial or total suppression of magnetic order, whose extent can be controlled by an applied field. We believe that the combination of the three themes of our study, namely neutron spectroscopy in quantum materials, magnetic field-induced phenomena and MPS methods of accessing the complete spectral response of arbitrary locally interacting spin models, offers an exciting near-term future for quantum magnetism.

## Methods

### Experimental information

High-quality single crystals of CsYbSe_2_ were prepared using the flux method^62^. Refinement of single-crystal X-ray diffraction data demonstrated the complete absence of Cs/Yb site mixing, as detailed in Supplementary Note 1A. For the characterization of our crystals, we measured the magnetization up to 60 T in pulsed magnetic fields at the National High Magnetic Field Laboratory (MagLab) and the specific heat in a dilution refrigerator at temperatures down to 0.05 K and magnetic fields up to 9 T (results shown in Supplementary Notes 1C and 1D). Our electron spin resonance measurements were performed using a continuous-wave ESR spectrometer, collecting data at X-band frequencies (*ν* = 9.4 GHz) and at *T* = 15 K. The resonance signal was measured from the field-derivative, *dP*/*dB* of the power, *P*, absorbed in a transverse microwave magnetic field and the spectra were fitted to a Lorentzian lineshape.

Approximately 200 single-crystalline pieces totaling around 0.5 g of material were co-aligned on copper plates to obtain a mosaic sample shown in Supplementary Fig. 4. Our neutron scattering experiments were performed on the time-of-flight (ToF) Cold Neutron Chopper Spectrometer (CNCS)^63^ at the Spallation Neutron Source at Oak Ridge National Laboratory (ORNL), the multiplexing Continuous Angle Multiple Energy Analysis spectrometer (CAMEA)^64^, and the cold-neutron Triple-Axis Spectrometer (TASP), the latter both located at the Swiss Spallation Neutron Source (SINQ) at the Paul Scherrer Institut (PSI). The measurements at CNCS were performed with an incident neutron energy *E*_i_ = 3.32 meV, providing an energy resolution of 0.11 meV. A cryomagnet equipped with a dilution refrigerator was used to provide a maximum magnetic field of *B* = 5 T at temperatures down to 0.07 K. Measurements at CAMEA were performed with incident neutron energies *E*_i_ = 5.2 and 6.2 meV (giving an energy resolution of 0.18 meV) and those at TASP with fixed *k*_i_ = *k*_f_ = 1.5 Å^−1^, both using an 11 T cryomagnet reaching a base temperature of *T* ≃ 0.02 K. In all three experiments, the sample was orientated in the (*HHL*) scattering plane, such that the vertical magnetic field was applied along the [−1 1 0] direction in the *ab* plane. The software packages MANTIDPLOT^65^ and HORACE^66^ were employed for the data reduction and analysis at CNCS, while the data collected at CAMEA were analysed with the MJOLNIR software package^67^.

### MPS calculations

We applied a cylinder MPS method to compute the dynamical spectral function of the isotropic spin-1/2 TLHAF in a magnetic field, as defined in Eq. (1), with *J*_2_/*J*_1_ = 0.03. The MPS method proceeds by computing the time-dependent spin-spin correlation function

2 $${C}_{{{{\bf{r}}}}}^{\alpha \beta }({{{\bf{x}}}},t)=\langle {\hat{S}}_{{{{\bf{r}}}}+{{{\bf{x}}}}}^{\alpha }(t){\hat{S}}_{{{{\bf{r}}}}}^{\beta }(0)\rangle ,$$

where **r** is the site at which the initial spin operator is applied, **x** is the vector separation in the two-point correlator, and *α*, *β* ∈ {*x*, *y*, *z*}. The cylinder size, the bond dimension of the matrices used in the representation and the time-evolution procedures required to obtain well converged spectral functions at all fields are discussed and benchmarked in Supplementary Note 5. The calculations were implemented in Python using the package TENPY^68^.

The dynamical spin spectral function was obtained from the Fourier transform

3 $${S}_{{{{\bf{r}}}},\alpha \beta }({{{\bf{Q}}}},\omega )=\int\nolimits_{-\infty }^{\infty }{{{\rm{d}}}}t\mathop{\sum}\limits_{{{{\bf{x}}}}}{e}^{i(\omega t-{{{\bf{Q}}}}\cdot {{{\bf{x}}}})}{C}_{{{{\bf{r}}}}}^{\alpha \beta }({{{\bf{x}}}},t).$$

The subscript **r** is retained because the correlation function is computed on a finite cylinder, with respect to the site **r** at its center, and by time-evolving a ground state that breaks the translational symmetry of the Heisenberg model. To restore this symmetry in the spectral function, we average over three distinct time-evolved states, each corresponding to a site in the central unit cell, as explained in Supplementary Note 5. This procedure offers a strong reduction of the computational cost when compared with the MPS calculation of time evolution for a single spatially symmetric state. To account for artifacts in the spectral function caused by the finite cylinder length and time series in the Fourier transform, we convolve ${C}_{{{{\bf{r}}}}}^{\alpha \beta }({{{\bf{x}}}},t)$ in Eq. (3) with a Gaussian envelope, as described in Supplementary Note 5. This results in an effective energy resolution of 0.1*J*_1_ ≡ 0.038 meV and a momentum resolution of 0.032/*a* ≡ 0.006 r.l.u.

For comparison with the measured INS data, the calculated components of the dynamical structure factor were converted into a cross-section using the relation

4 $$\frac{{d}^{2}\sigma }{d\Omega d\omega }\,\propto \,\,| F({{{\bf{Q}}}}){| }^{2}\,\mathop{\sum}\limits_{\alpha ,\beta }\,\left(\,{\delta }_{\alpha \beta }-\frac{{Q}_{\alpha }{Q}_{\beta }}{{Q}^{2}}\,\right)\,{({g}_{\alpha \beta })}^{2}{S}_{\alpha \beta }({{{\bf{Q}}}},\omega ),$$

where *F*(**Q**) is the magnetic form factor of the Yb^3+^ ion, (*δ*_*αβ*_ − *Q*_*α*_*Q*_*β*_/*Q*^2^) is the neutron scattering polarization factor and *g*_*αβ*_ specifies the components of the *g*-tensor determined by ESR. A detailed comparison of our INS and MPS results is shown in Supplementary Note 6.

### Supplementary information

Supplementary Information to accompany the article

## Data Availability

The data that support the findings of this study are available from the corresponding author upon reasonable request.

## Code availability

The code that supports the findings of this study is available from the corresponding author upon reasonable request.
