# Supplementary material for: Complete field-induced spectral response of the spin-1/2 triangular-lattice antiferromagnet CsYbSe2
Source: NPJ Quantum Mater. 2023 Sep 23;8(1):48. doi: 10.1038/s41535-023-00580-9 (PMC11041694; doi:10.1038/s41535-023-00580-9)
Supplement: Supplementary file 1 — Supplementary Information to accompany the article [file 41535_2023_580_MOESM1_ESM.pdf]

Supplementary Information to accompany the article
"Complete field-induced spectral response of the spin-1/2 triangular-lattice antiferromagnet $CsYbSe_2$"

Tao Xie, A. A. Eberharter, Jie Xing, S. Nishimoto, M. Brando, P. Khaneko, J. Sichelschmidt, A. A. Turrini, D. G. Mazzone, P. G. Naumov, L. D. Sanjeewa, N. Harrison, Athena S. Sefat, B. Normand, A. M. Läuchli, A. Podlesnyak and S. E. Nikitin

Supplementary Note 1. CHARACTERIZATION

A. Single-Crystal X-Ray Diffraction

We have performed a careful investigation of the lattice structure of our $CsYbSe_2$ samples by analyzing several batches of single crystals using a Bruker Quest D8 single-crystal X-ray diffractometer. The structure was refined by the Rietveld method using the FullProf software package [1], which delivered excellent structural solutions with no evidence for site mixing. In these refinements, we treated the anisotropic displacement parameters and site occupancies as free variables, and in this way confirmed the complete absence of site disorder. The crystallographic data are presented in Supplementary Table 1, and the result of a Rietveld refinement is shown in Supplementary Figure 1; we have also reported this crystal structure in the Cambridge Crystallographic Data Centre (CCDC) [2]. In addition we found no significant residual electron density, which would have suggested the presence of interstitial atoms. All of these results confirmed the high quality of our single crystals.

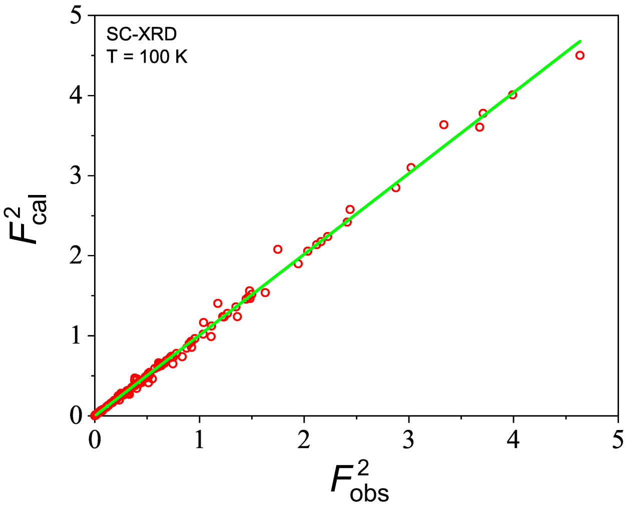

Supplementary Figure 1. Rietveld refinement of single-crystal X-ray diffraction data at 100 K. F^2_{obs} and F^2_{cal} denote respectively the observed and calculated structure factors.

B. Crystal Structure and Next-Neighbour Magnetic Interaction

One of the key questions in the analysis of every compound in the Yb-delafossite family is whether the ground state of the $S = 1/2$ system can be a quantum spin liquid (QSL) at zero applied magnetic field. In our work we have confirmed the presence of 120° order in $CsYbSe_2$, at temperatures below 0.4 K and at least over a spatial range far exceeding the lattice constant [Fig. 1c of the main text and Supplementary Figure 6(e) below]. The same order is found in $KYbSe_2$ [3], but notably not in $NaYbSe_2$ [4]. Factors destabilizing this order include a possible frustrated coupling between triangular-lattice (TL) planes and a possible next-neighbour interaction within the planes, where (as noted in the main text) recent numerical studies have achieved partial agreement that the ground state is a QSL in the regime $0.06 \lesssim J_2/J_1 \lesssim 0.15$ [5–10].

Addressing first the issue of layer stacking, the majority of Yb delafossites have a structure described by the $R\bar{3}m$ space group, as listed in Supplementary Table 2, which has an ABC stacking that does indeed suggest frustration of antiferromagnetic (AF) interlayer couplings. However, some members of the series with larger alkali-metal ions, including Cs (Supplementary Table 2), have a structure with space group $P6_3/mmc$ that has AA stacking, and hence no interlayer frustration. Thus the available materials examples tend to suggest that the stacking of TL layers is not a relevant factor. Turning to J_2, in our work we have deduced a weak next-

Supplementary Table 1. Crystallographic data for $CsYbSe_2$ determined by single-crystal X-ray diffraction.

Empirical Formula	$CsYbSe_2$
Formula weight (g/mol)	463.87
T, K	100
Crystal habit	red plates
Crystal dimensions, mm	$0.14 \times 0.10 \times 0.03$
Crystal system	hexagonal
Space group	$P6_3$/mmc (No. 194)
a, Å	4.1466(2)
c, Å	16.5050(1)
Volume, Å^3	245.77(3)
Z	2
Density (calc), g/cm^3	6.268
$\mu(\mathrm{MoK}_\alpha)$, mm^{-1}	40.932
F(000)	386
T_{max}, T_{min}	0.4415, 1.0000
θ range for data collection	2.47-29.93
Reflections collected	3895
Final R $[I>2\sigma(I)] R_1, R_{w2}$	0.0274/0.0833
Final R (all data) R_1, R_{w2}	0.0284/0.0843
Goodness of fit, F^2	1.343

Supplementary Table 2. Crystallographic information for selected Yb-selenide delafossites [11].

Chemical formula	$NaYbSe_2$	$KYbSe_2$	$RbYbSe_2$	$CsYbSe_2$
Crystal system	trigonal	trigonal	trigonal	hexagonal
Space group	R$\bar{3}m$ (No. 166)	R$\bar{3}m$ (No. 166)	R$\bar{3}m$ (No. 166)	$P6_3/mmc$ (No. 194)
Stacking type	ABC	ABC	ABC	AA
Ionic radius, Å	1.02 (Na^+)	1.38 (K^+)	1.52 (Rb^+)	1.67 (Cs^+)
Interlayer distance, Å	6.92	7.56	7.88	8.25

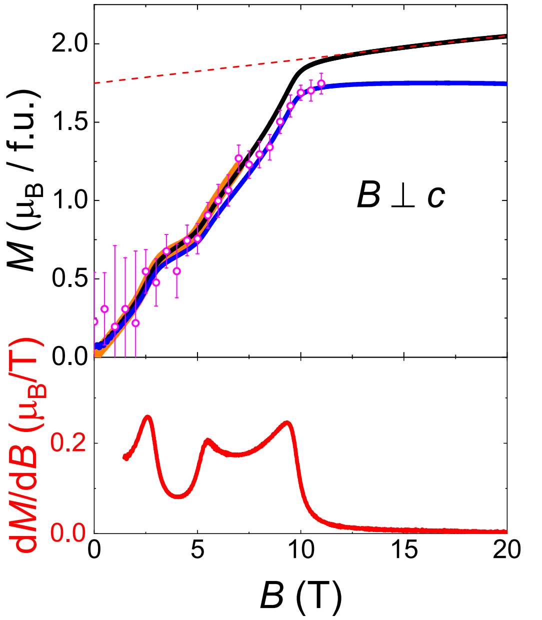

Supplementary Figure 2. Isothermal magnetization measured in a pulsed magnetic field. The black curve shows the raw magnetization data, the red dashed line the estimated van Vleck contribution and the blue curve the intrinsic magnetization obtained by subtraction of the van Vleck part. The orange symbols are the low-field (up to 7 T) magnetization data measured in a MPMS-7. Open circles show the bulk moment extracted from the field-dependence of the (0, 0, 4) Bragg peak measured by neutron diffraction at $T < 0.05$ K. The solid red curve in the lower panel shows the first derivative of the magnetization, dM/dB.

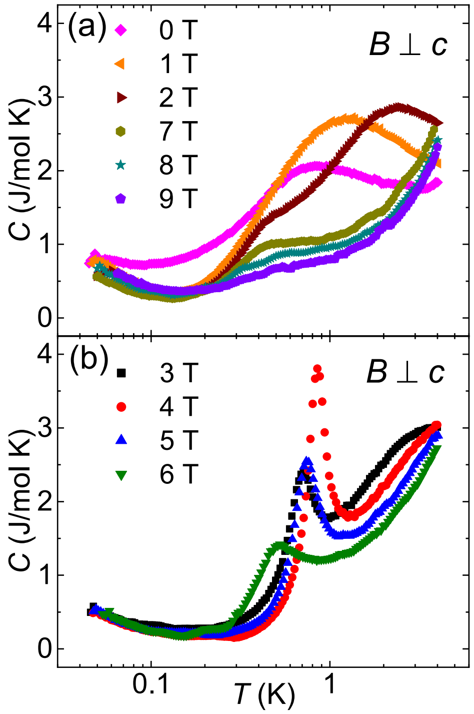

Supplementary Figure 3. Temperature-dependence of the specific heat measured in a range of applied in-plane magnetic fields ($\mathbf{B} \perp \hat{c}$).

neighbour interaction in $CsYbSe_2$, $J_2 = 0.03J_1$. The authors of Ref. [3] deduced a value $J_2 = 0.05J_1$ in $KYbSe_2$, placing it closer to the QSL regime. While the authors of Ref. [4] were not able to determine a J_2 value, one may certainly suggest that the larger lattice constant inherent to the members with larger alkali-metal ions (Supplementary Table 2) causes a reduction of J_2 and hence an increasing stabilization of the 120°-ordered ground state at base temperature in the Yb-delafossite materials.

C. Magnetization in Pulsed Magnetic Field

We measured the isothermal magnetization at $T = 0.4$ K in pulsed magnetic fields up to 60 T, and for calibration in a MPMS-7 with a ^{3}He insert at fields up to 7 T. We note that the magnetic field in all our experiments is applied in the ab plane, unless otherwise stated, and that we do not distinguish between in-plane directions. In the raw magnetization data shown by the black solid line in Supplementary Figure 2, we observe a clear and continuous increase above a saturation field of approximately 10 T. This contribution results from van Vleck paramagnetism and can be approximated in lowest order by a linear function (the dashed red line), from which the van Vleck susceptibility may be estimated as $\chi^{VV} \approx 0.0152\ \mu_B$/T. Subtracting this contribution from the raw data [12–14] leaves an intrinsic magnetization [solid blue line in Supplementary Figure 2, shown also in Fig. 1c of the main text] displaying near-perfect saturation above a field B_{Sat} that we estimate most accurately from the excitation spectrum of the fully polarized phase [Fig. 2b of the main text]. In Supplementary Figure 2 we show also the bulk magnetization obtained from the field-induced enhancement of the (0 0 4) Bragg peak measured on TASP at the Paul Scherrer Institute (PSI), and these results agree quantitatively with the di-

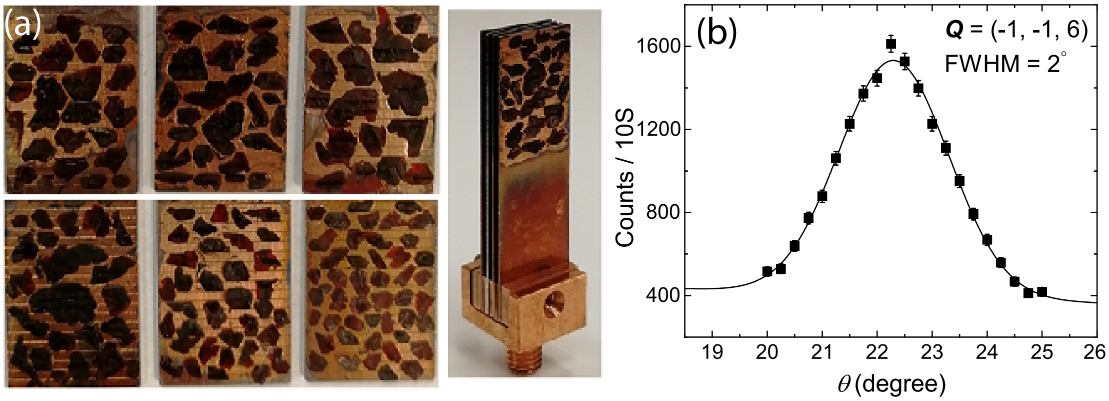

Supplementary Figure 4. (a) Photographs of the $CsYbSe_2$ crystals coaligned and assembled for neutron scattering experiments. (b) Rocking curve of the coaligned sample measured by neutron diffraction, with a Gaussian fit shown by the solid line.

rect measurements. We defer the accurate modelling of these data, which we performed to obtain the orange line in Fig. 1c of the main text, to Supplementary Note 4.

D. Specific Heat

We measured the specific heat in a dilution refrigerator at temperatures down to 50 mK and magnetic fields up to 9 T. A sharp peak in $C(T)$ can be used to establish the presence of long-range order in the system. In Supplementary Figure 3(a) we group the applied fields, namely those below 3 T and above 6 T, in which no clear peak appears in $C(T)$ at all, only a broad hump at the low fields and a weak shoulder at the high fields. In Supplementary Figure 3(b) we show that relatively sharp, λ-shaped peaks can be found at 3, 4 and 5 T, in partial correspondence with the long-range order of the UUD phase found by neutron diffraction. The temperatures of these sharp peaks are indicated as solid circles in the phase diagram shown in Fig. 1d of the main text and the temperatures of the features found at $B \leq 2$ T and $B \geq 6$ T as open circles.

In more detail, the specific heat at zero field in Supplementary Figure 3(a) shows a broad hump with no trace of a phase transition. This should be contrasted with the neutron diffraction measurements in Fig. 1b of the main text, which display a series of weak magnetic Bragg peaks with a clear onset at $T \simeq 0.4$ K. However, the analysis of the correlation lengths presented in Supplementary Note 2B leads to the result that these are finite at zero field, signalling a lack of true, long-ranged AF order even at $T = 0.02$ K, which is consistent with the absence of a sharp peak in the corresponding $C(T)$ data. The specific heat does show sharp, λ-shaped peaks in the field range 3-5 T [Supplementary Figure 3(b)] that reflect phase transitions into a long-range-ordered phase on, and apparently near, the 1/3-plateau state. These thermodynamic data are confirmed by the field-dependence of the (1/3, 1/3, 1) magnetic peak presented in Fig. 1c of the main text and analyzed in detail in Supplementary Figure 7 below.

Supplementary Note 2. NEUTRON SCATTERING EXPERIMENTS

A. Sample Preparation

We coaligned around 200 single crystallites on copper plates, as shown in Supplementary Figure 4(a), to obtain a mosaic sample with a total mass of approximately 0.5 g. The rocking curve obtained by a neutron diffraction measurement of one selected magnetic peak [Supplementary Figure 4(b)] shows that the mosaicity of this sample is approximately 2° FWHM.

B. Elastic Neutron Scattering at CNCS

Supplementary Figure 5 shows two-dimensional (2D) slices through the measured intensity dataset at zero energy in the $(H\ H\ L)$ plane at fields from 0 to 5 T. At 0 T we observe weak but nonetheless clear intensity peaks at $Q = (1/3,\ 1/3,\ L)$ for odd-integer L, which become weaker and almost invisible at 1 T. For $B \geq 2$ T we find rods of magnetic intensity elongated along the $[1/3\ 1/3\ L]$ direction, with peaks at the same Q values. The intensities of these peaks signal effectively long-ranged order around 3-5 T, their very broad nature in L reflects the 2D nature of the magnetic system (as opposed to new peaks at intermediate L values) and their $(H,\ K)$ position confirms the persistence of threefold periodicity in the plane of the triangular lattice at all applied fields. In the context of Supplementary Figure 5(a), we state for completeness that the data shown in the upper panel of Fig. 1b of the main text were integrated over the interval $K = [-0.05, 0.05]$, symmetrized according to the crystal symmetry and unfolded for visual clarity, meaning that the intensities at $\pm L$ are equivalent.

For a specific magnetic peak, the spin correlation length can be estimated using the formula $\xi = 2\pi/\sqrt{w^2 - R^2}$ [15], where w is the full width at half maximum (FWHM) height of the magnetic peak and R is the instrumental momentum resolution at this peak. To estimate this resolution, we first

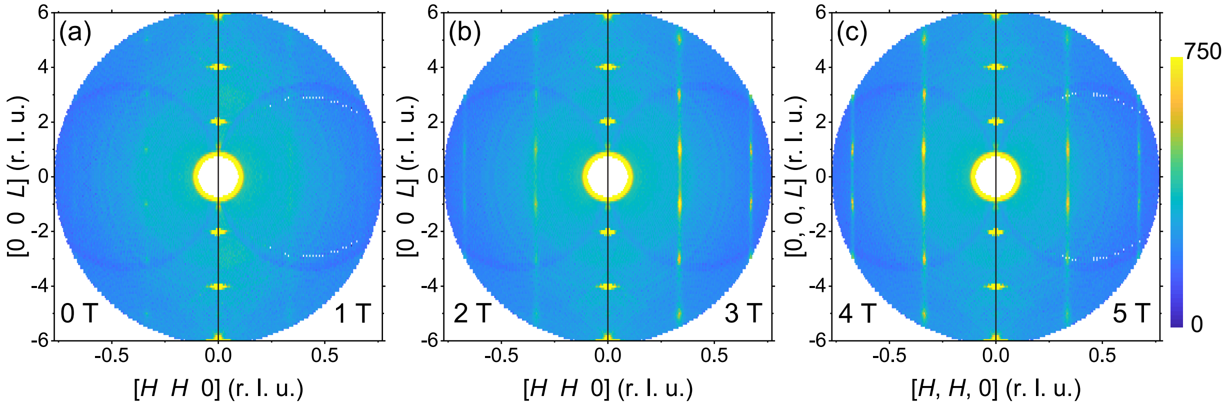

Supplementary Figure 5. Constant-energy slices in the $(H\ H\ L)$ plane taken at zero energy transfer under different magnetic fields, with the data symmetrized about the $[H\ H\ 0]$ axis. The strong spots at $Q = (0,\ 0,\ L)$ for even-integer L are nuclear Bragg peaks.

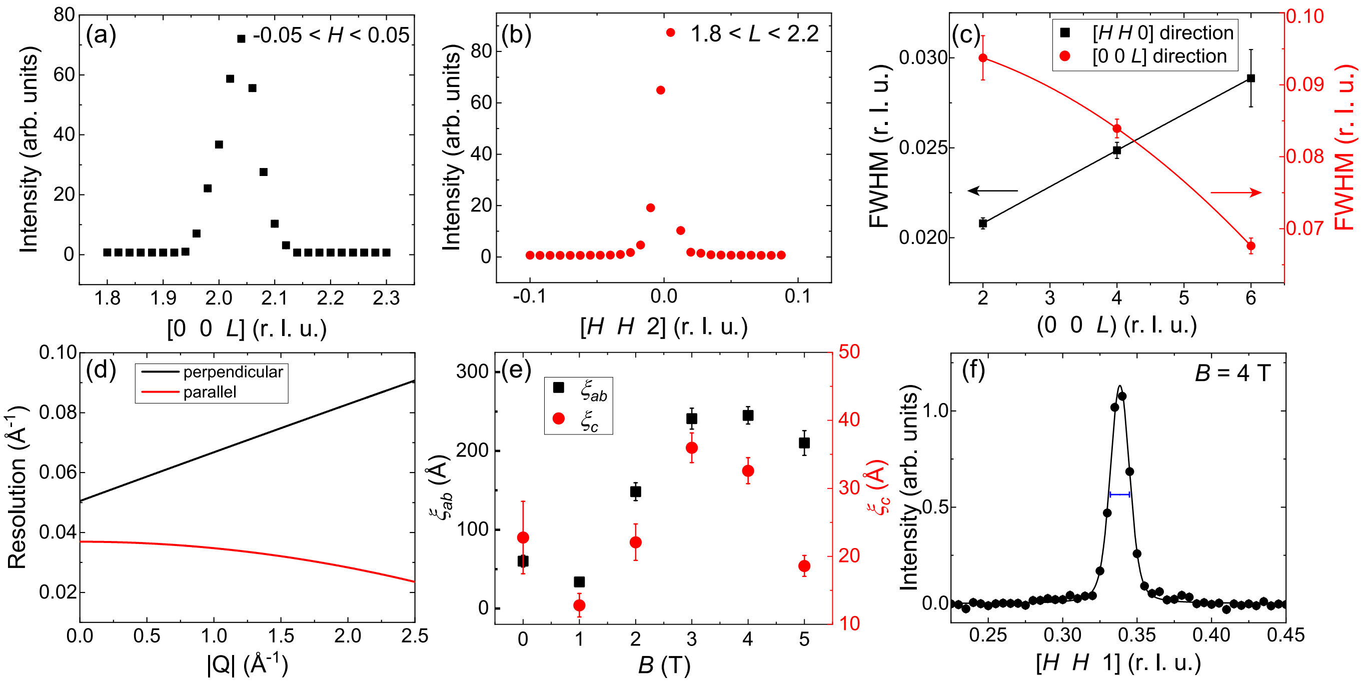

Supplementary Figure 6. (a,b) 1D intensity cuts at zero energy along the $[0\ 0\ L]$ and $[H\ H\ 2]$ directions at the (0, 0, 2) nuclear Bragg peak at 0 T. (c) FWHM of nuclear peaks obtained from cuts along the $[H\ H\ 0]$ direction (black) and $[0\ 0\ L]$ direction (red) at Q = (0, 0, 2), (0, 0, 4) and (0, 0, 6). Solid lines in both panels show polynomial fits. (d) Perpendicular and parallel $|Q|$-resolution estimated from the FWHMs shown in panel (c). (e) Perpendicular ($\xi_\perp$) and parallel ($\xi_\parallel$) correlation lengths calculated from the magnetic Bragg peaks shown in Supplementary Figure 5. For this geometry $\xi_\perp \equiv \xi_{ab}$, the in-plane correlation length, and $\xi_\parallel \equiv \xi_c$, the out-of-plane correlation length. (f) An example 1D cut along the $[H\ H\ 1]$ direction through the magnetic Bragg peak (1/3, 1/3, 1) at 4 T. The horizontal blue bar represents the instrumental $|Q|$-resolution, which indicates a resolution-limited peak at 4 T.

prepared 1D intensity cuts along the $[H\ H\ 0]$ and $[0\ 0\ L]$ directions at the nuclear peaks (0, 0, 2) [Supplementary Figure 6(a,b)], (0, 0, 4) and (0, 0, 6), then fitted a Gaussian function to them. The FWHM values of these three nuclear peaks reflect the momentum resolution of the instrument perpendicular (for $[H\ H\ 0]$) and parallel (for $[0\ 0\ L]$) to $\hat{Q}$ at each reciprocal-lattice point. Polynomial fits to the FWHM as a function of L [Supplementary Figure 6(c)] yield approximate estimates of the resolution as a function of $|\mathbf{Q}|$ for an arbitrarily chosen momentum transfer, $\mathbf{Q}$, in the perpendicular and parallel directions [Supplementary Figure 6(d)].

To obtain the FWHM of the magnetic peaks for a quantitative determination of the correlation length, we fitted 1D cuts through the magnetic peaks to the Voigt function, which is defined as

$$y(x) = y_0 + (f_1 * f_2)(x), \tag{1}$$

where the $*$ denotes a convolution. In Eq. (1), $f_1(x) =$

$\frac{2A}{\pi}\frac{w_L}{4(x-x_c)^2+w_L^2}$ is the Lorentz function, with peak centre x_c and FWHM w_L, and $f_2(x) = \sqrt{\frac{4\ln 2}{\pi}}\frac{1}{w_G}\exp(-\frac{4\ln 2}{w_G^2}x^2)$ is a Gaussian with peak centre $x = 0$, unit peak area and FWHM w_G, which should be fixed to the instrumental resolution, $R(|\mathbf{Q}|)$, for a specific $\mathbf{Q}$. The FWHM of the Voigt function is then $w_V = 0.5346 w_L + \sqrt{0.2166 \cdot w_L^2 + w_G^2}$. By inserting w_V for w and $R(|\mathbf{Q}|)$ for R into the above expression for ξ, we estimate the correlation lengths at $\mathbf{Q}$ = (1/3, 1/3, 1) for each different field. Supplementary Figure 6(e) shows the correlation lengths we deduce from all of our CNCS data, which from our choice of the (0 0 L) series of nuclear peaks correspond to the in- and out-of-plane correlations. At $B = 0$ we obtain the values $\xi_{ab} = 60(7)$ Å and $\xi_c = 23(5)$ Å quoted in the main text. We observe that the correlation lengths dip at 1 T before ξ_{ab} rises strongly, to values in excess of 200 Å, on and just above the 1/3 plateau. To gauge the meaning of this number, in Supplementary Figure 6(f) we show a 1D cut along $[H\ H\ 1]$ at $B = 4$ T: the horizontal blue bar is the $|Q|$-resolution at the magnetic Bragg peak (1/3, 1/3, 1), which is clearly resolution-limited, and thus the intrinsic ξ values diverge as expected for the long-range-ordered UUD state.

C. Elastic Neutron Scattering at TASP

The dependence of the integrated intensity of the magnetic peak (1/3, 1/3, 1) on the applied magnetic field is summarized in Fig. 1c of the main text and its dependence on temperature in Fig. 1b. Supplementary Figure 7(a) shows the raw elastic neutron scattering data measured on TASP in scans along the $[H\ H\ 1]$ direction at base temperature for a series of fields. The peak intensity is clearly suppressed by a weak field, with a minimum around 1 T, before becoming sharper and stronger as the field is increased, reaching a maximum around 4 T. This intensity then decreases beyond 5 T to very small values higher in the V regime. Supplementary Figure 7(b) shows data obtained for the same scan at zero field for multiple temperatures below 1 K, where the robust peak present at $T = 0.35$ K clearly becomes sharper and stronger towards base temperature.

D. Two-Dimensional Excitation Character

The spin excitations we observe in our measurements retain their highly 2D nature over the full energy range and under all applied fields. In the representative 2D constant-energy slices presented in Supplementary Figure 8, the intensity distribution in all cases takes the form of multiple rods extending largely unchanged along the L direction. This definitive proof of 2D spin excitations is fully consistent with previous reports on $CsYbSe_2$ [16] and related delafossites [3, 4]. As a consequence we may integrate our intensity data over a wide L range to analyse the in-plane spin dynamics, and for the data shown both below and in Fig. 3 of the main text, this integration range, $1.2 \leq L \leq 3.8$, is denoted by the L label 2.5.

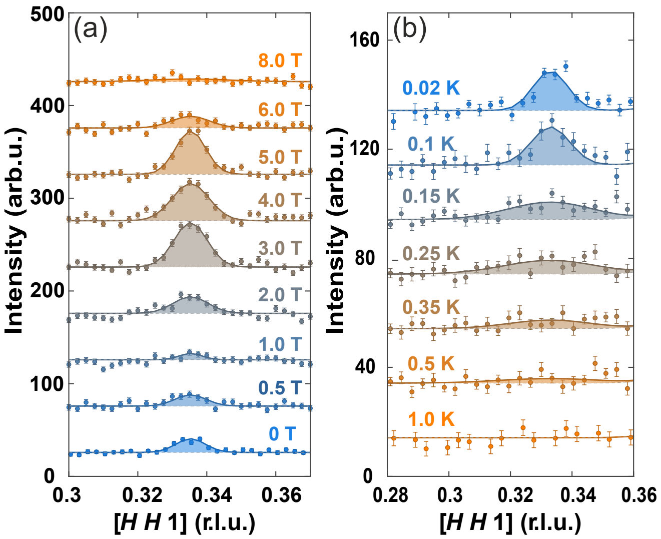

Supplementary Figure 7. (a) Intensity of the (1/3, 1/3, 1) magnetic Bragg peak measured at $T < 0.05$ K for a series of magnetic fields, which are offset by 50 units for clarity. Solid lines and shading show Gaussian fits. (b) Intensity of this peak measured at zero field for a series of temperatures below 1 K, which are offset by 20 units for clarity.

E. Background Definition and Subtraction for INS Spectra

The INS spectrum is always contaminated by a background contribution that arises from incoherent neutron scattering and from scattering due to the sample environment. Raw spectral data obtained on CNCS for the $[H\ H\ 2.5]$ direction (meaning with the broad L integration described above) are shown in the left panels of Supplementary Figure 9(a,c,d) for three different magnetic fields at our base temperature of 70 mK. For an accurate characterization of the background, we make use of the fact that the 5 T spectrum at the Γ point, $I(\mathbf{Q} = \mathbf{0}, E)$, consists of a single and well-defined inelastic peak near 1 meV. We assume that an appropriate 1D intensity cut, prepared from the shaded region in Supplementary Figure 9(a) and shown by the black points in Supplementary Figure 9(b), can be described within the energy window 0.5 meV $\leq E \leq$ 1.5 meV by a Lorentzian peak and a linear background. Thus we fitted the measured signal to the form

$$I(E) = a_0 + a_1 E + \frac{I_0 W_0^2}{(E - E_0)^2 + W_0^2}, \qquad (2)$$

where I_0, W_0 and E_0 characterize respectively the intensity, width and centre of the inelastic peak. We subtract the fitted Lorentzian, shown by the blue line in Supplementary Figure 9(b), from the raw spectrum to obtain a residual intensity that we consider as fully representative of the background [shown by the red points in Supplementary Figure 9(b)]. The results of subtracting this background are shown in the right panel of Supplementary Figure 9(a), where clear limits to the extent of each continuum become visible. Because our data provide no evidence that the background varies with $\mathbf{Q}$, we subtracted the same form from our cuts everywhere in reciprocal space.

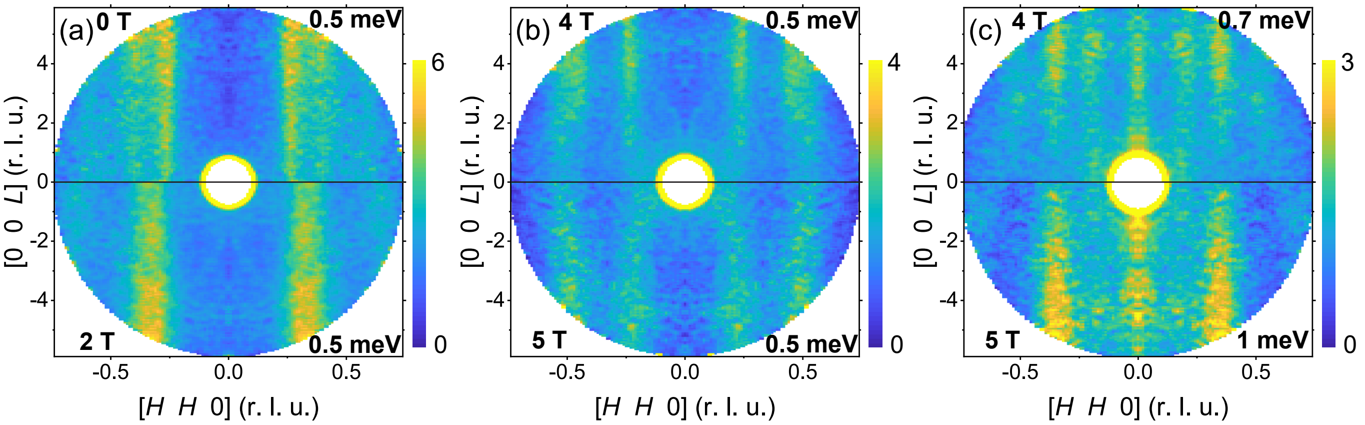

Supplementary Figure 8. Constant-energy 2D intensity slices at different energies and magnetic fields. The data have been symmetrized about the [0 0 L] axis.

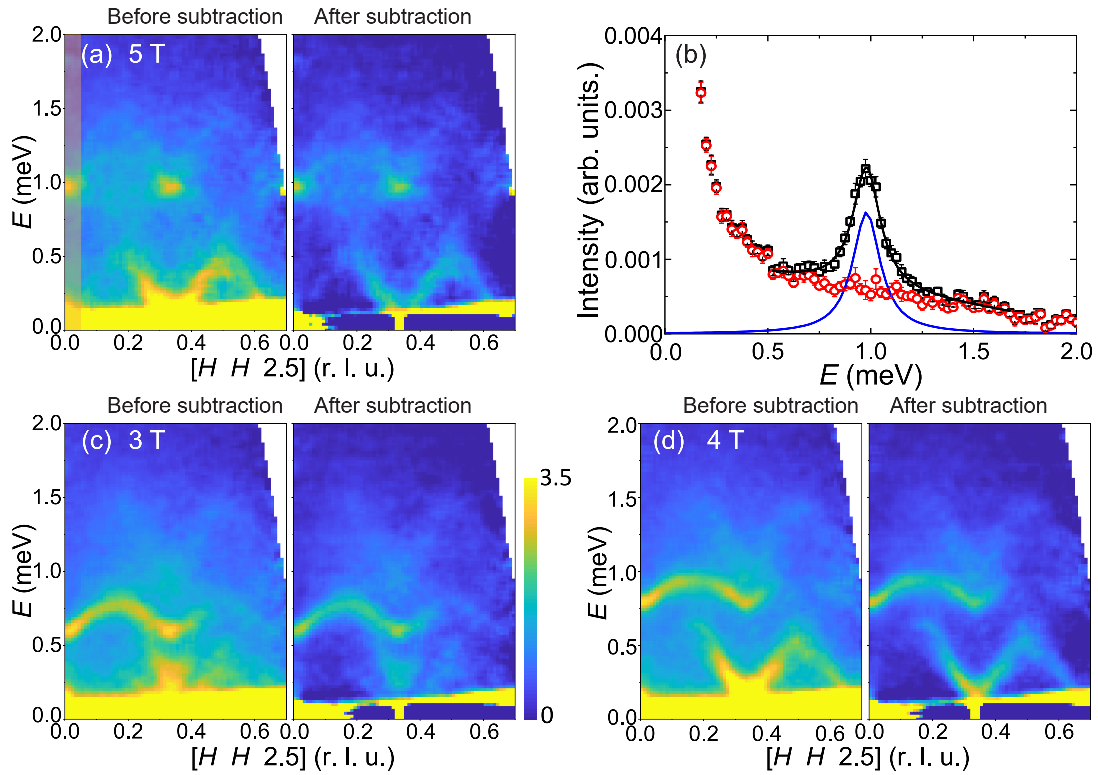

Supplementary Figure 9. Definition of the subtracted background in the INS spectra and illustration of selected INS spectra before and after background subtraction. (a) Left: raw data for the spin excitation spectrum along $[H\ H\ 2.5]$ at 5 T and 70 mK; right: corresponding spectrum obtained after background subtraction. This background is extracted from the shaded area ($0 \leq H \leq 0.05$). (b) Open black squares show the 1D energy cut obtained by integrating over the shaded region in panel (a). The solid black line shows a Lorentz fit to this cut with a linear background, the blue line shows the Lorentz peak fit alone, and the open red circles show the difference, which is used as the real background for subtraction purposes. (c) INS spectra before and after background subtraction for $B = 3$ T. (d) Spectra before and after subtraction for $B = 4$ T.

Similarly, the background has no dependence on the magnetic field, and examples of full background-subtracted spectra at $B = 3$ and 4 T are shown in Supplementary Figure 9(c,d). We stress that this method provides a minimal parameter-free background model, and its simplicity far outweighs the disadvantage of a minor (statistically insignificant) oversubtraction appearing at some high H values.

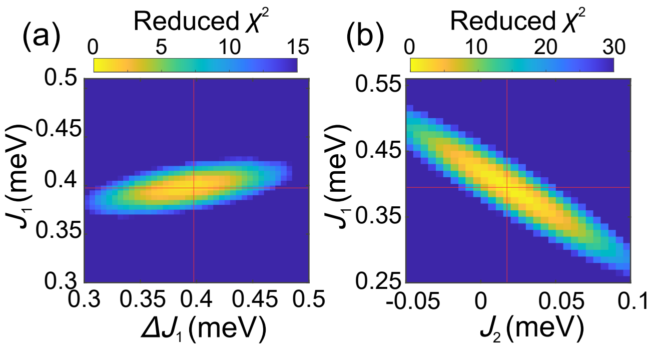

Supplementary Figure 10. (a) Fit quality shown as a function of assumed XXZ-model parameters J_1 and Δ for the dominant nearest-neighbour interaction. Here J_2 has been fixed to its optimal value of 0.011 meV. (b) Fit quality shown as a function of J_1 and J_2, assuming $\Delta = 1$. Red crosses in both panels indicate the position of the global minimum.

Supplementary Note 3. LINEAR SPIN-WAVE THEORY

In our analysis we use linear spin-wave theory (SWT) to achieve two separate goals. Working above the saturation field, B_{Sat}, we make use of the fact that the excitations are guaranteed to be well defined spin waves to obtain the most accurate available fit of the magnetic interaction parameters of $CsYbSe_2$ (Supplementary Note 3A). Working below B_{Sat}, we use linear SWT for a preliminary indication of the locations and energy scales of putative $\Delta S = 1$ excitations in the TLHAF, and hence of departures from semiclassical magnetism arising due to quantum corrections. In Supplementary Note 3B we outline how the SWT results shown in Fig. 3 of the main text were obtained and use these to obtain an overview of possible two-magnon contributions.

A. Fitting of Magnetic Interactions

As described in the main text, we determine the magnetic interactions by using the 11 T dataset from CAMEA, which shows a single, sharp magnon mode with its maximum at the Γ point [Fig. 2b of the main text]. We quantified the location of this mode at 17 points in reciprocal space and used SpinW [17] to fit these to the Hamiltonian

$$\mathcal{H} = J_1 \sum_{\langle i,j\rangle} (S_i^x S_j^x + S_i^z S_j^z + \Delta S_i^y S_j^y) + J_2 \sum_{\langle\langle i,j\rangle\rangle} \mathbf{S}_i \cdot \mathbf{S}_j - \mu_B g_{ab} B \sum_i S_i^z. \quad (3)$$

The optimal fit in the 3D parameter space of J_1, Δ and J_2 is $J_1 = 0.395(7)$ meV, $\Delta J_1 = 0.39(2)$ meV and $J_2 = 0.011(4)$ meV. In Supplementary Figure 10 we illustrate the quality of this fit by showing two 2D cross-sections: fixing J_2 to its optimal value [Supplementary Figure 10(a)] shows that $J_1 = \Delta J_1$, i.e. the nearest-neighbour interaction is isotropic within the precision of our measurements; setting $\Delta = 1$ [Supplementary Figure 10(b)] allows us to optimize J_2, and hence to conclude that magnetic Hamiltonian of $CsYbSe_2$ is described by the J_1-J_2 Heisenberg model with $J_1 = 0.395(8)$ meV and $J_2 = 0.011(4)$ meV ($J_2/J_1 = 0.03$).

B. SWT Spectra

Linear SWT ceases to provide a complete description of the spectrum of the TLHAF as soon as the field is lowered below B_{Sat}, when quantum corrections become finite. The TLHAF in an in-plane magnetic field has long been known [18] to exhibit a deformed 120° phase (Y), the 1/3 plateau phase (UUD) and a V (or 2:1) phase, with two of the spins parallel, below saturation, as illustrated schematically in Fig. 1d of the main text. At the mean-field level, the magnetization is perfectly linear in field from 0 to $B_{\mathrm{Sat}} = 9.6$ T (Fig. Supplementary Figure 12), which is obtained exactly due to the classical nature of the problem at $B \geq B_{\mathrm{Sat}}$. However, $M(B)$ has no plateau around $B_{\mathrm{Sat}}/3$, as this phase is stabilized only at higher order in $1/S$. In linear SWT, there is a large family of classically degenerate states at any field $B < B_{\mathrm{Sat}}$, and the precise orientation of the Y, UUD or V state at any given field is set manually in our analysis. The spin excitations around this fixed ground state were then calculated using SpinW and the resulting spectra at all selected fields in our experimental range ($B =$ 0-11 T) are shown as the orange lines in Fig. 3 of the main text.

For completeness, in Supplementary Figure 11 we show the densities of two-magnon states computed using the linear SWT one-magnon branches at fields of 0, 2, 3, 4, 5 and 8 T. The full two-magnon spectral functions are reweighted versions of these densities of states, which for the Y and V phases are more complex to compute and to normalize to the one-magnon branches. The density of states therefore serves as a useful semi-quantitative guide to the location of the two-magnon continuum in wavevector and energy, and to its qualitative shape. The densities of states in Supplementary Figure 11 each reflect the nine different continua arising from the three one-magnon branches, whose overlap results in the edge structures that appear throughout the Brillouin zone. The primary difference between linear SWT and our INS and MPS results is clearly the absence of well defined one-magnon branches extending over most of the zone at all fields outside the UUD phase. While the two-magnon continua of linear SWT occupy the entire zone over a broad energy window centred at 1 meV, they are in general too flat (except at 8 T), too uniform and have too many edges to bear a close resemblance to the continuum features in our measured and calculated spectral functions.

Supplementary Note 4. MAGNETIZATION RESPONSE OF THE TLHAF

To interpret the magnetic response despite the effects of the finite-temperature rounding, we estimate B_{Sat} from the TLHAF model parameters obtained by high-field INS (Sup-

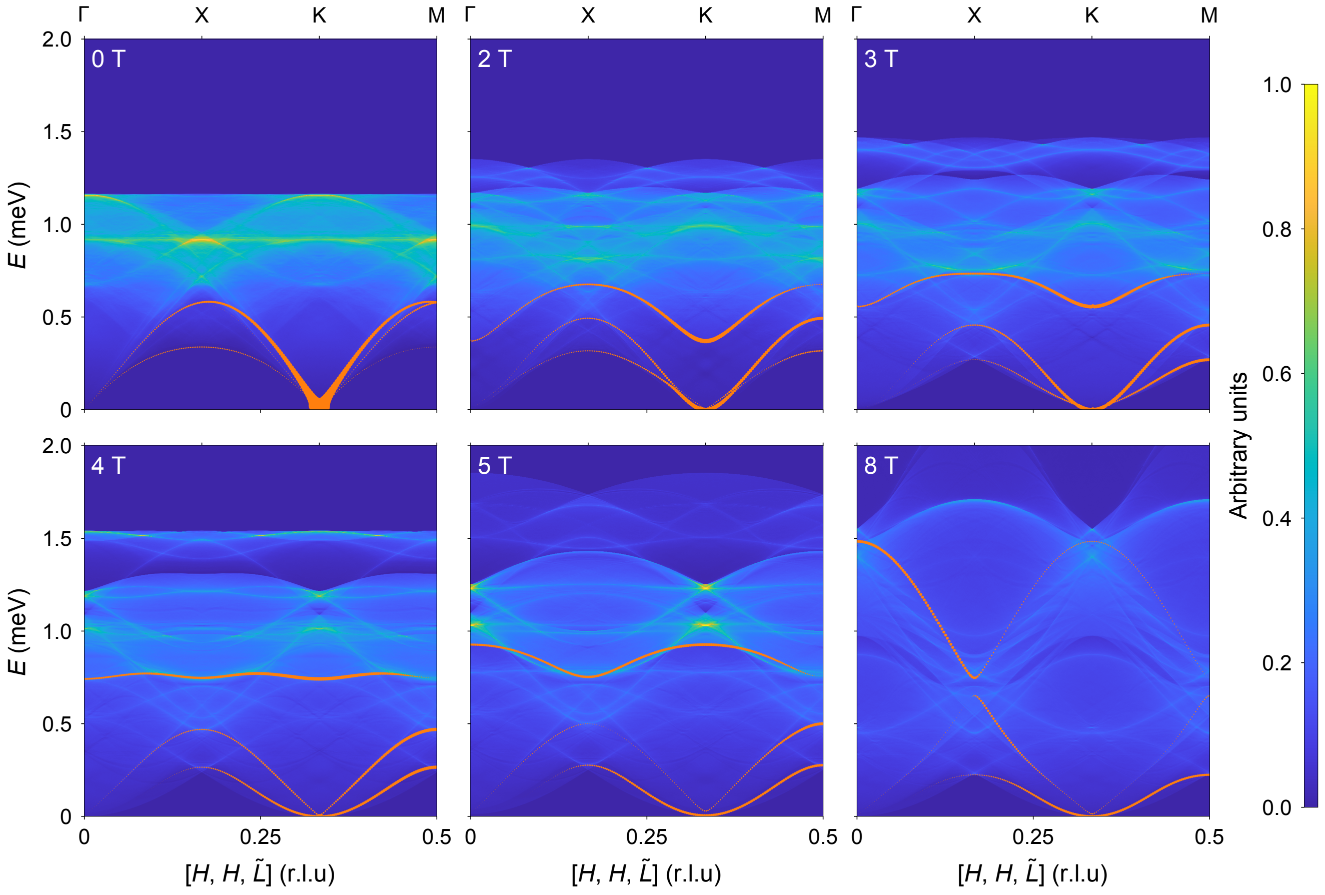

Supplementary Figure 11. Two-magnon densities of states computed from linear SWT at six different magnetic fields for the J_1-J_2 TLHAF with $J_2/J_1 = 0.03$. $\tilde{L}$ denotes integration over the full range of L, equivalent to an ideally two-dimensional system.

plementary Note 3A) and perform a grand canonical DMRG calculation of the full magnetization curves that allows us to deduce the boundaries of the 1/3-magnetization plateau.

For a state-of-the-art numerical determination of the magnetization response of the $S = 1/2$ TLHAF, we apply the grand canonical DMRG method [19]. This technique computes the infinitesimally small magnetization response to a change in the applied field, and thus the physical quantities it provides mimic the thermodynamic limit to approximately 1 part in 10^3 for a 2D system. The method is based on a graded division of a finite-sized cluster into a centre region and edge regions, such that the centre reproduces the continuous bulk response by using the near-zero-energy states of the edge as a type of buffer. In a system of fixed size and shape, the grading is introduced by modulating the energy scale with the externally imposed function

$$f(r) = \frac{1}{2}\left[1 + \cos\left(\frac{\pi r}{R}\right)\right], \qquad (4)$$

which deforms the Hamiltonian smoothly from its standard energy at the centre of the system ($r = 0$) to zero at the open cluster edges ($r = R$). After obtaining the lowest eigenwavefunction of the deformed Hamiltonian, the magnetization can be read as $M = \langle S^z(r = 0)\rangle$, because this wavefunction optimizes the expectation value to its thermodynamic limit at any given magnetic field. To mimic the results of a bulk measurement, appropriately weighted for the different possible directions of the magnetic field with respect to the triangular lattice, we performed our calculations using the hexagonal 75-site cluster shown in the inset of Supplementary Figure 12. The calculations were performed for the J_1-J_2 TLHAF using the interaction parameters deduced in Supplementary Note 3A and an in-plane g-factor of $g_{ab} = 3.2$, as deduced from ESR and INS in the main text. The result shown in Supplementary Figure 12 is that reproduced in Fig. 1c of the main text, and was used for our extraction of the lower and upper boundaries ($B_{\rm l}$ and $B_{\rm u}$) of the 1/3 plateau.

Supplementary Note 5. MPS CALCULATIONS

The wavefunction of the spin system is represented by a matrix-product state (MPS), which assigns a tensor to each lattice site. The accuracy of the MPS approximation is controlled by the maximum bond dimension, χ, of these tensors. To study the TLHAF we tested values of this parameter up to $\chi = 1024$, and will illustrate its role below. We used the Python package TenPy [20], specifically its algorithms for the

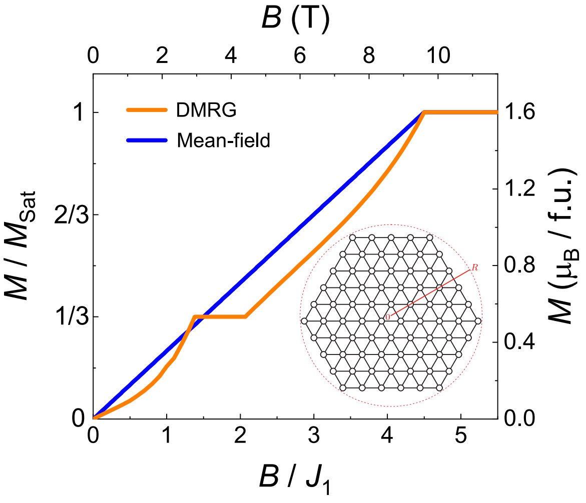

Supplementary Figure 12. Magnetization, $M(B)$, of the J_1-J_2 TLHAF with J_2/J_1 = 0.03 calculated by grand canonical DMRG and compared with the mean-field result. The inset shows the 75-site open cluster used for the DMRG calculation.

ground state and for time-evolution, as well as its support for preserving the U(1) symmetry of a model.

We start by describing the cylinder geometry used for MPS calculations. We implement a cylinder with circumference C, length L and the "XC" boundary conditions shown in Supplementary Figure 13(a) [3, 21]. This geometry provides a high momentum resolution along the Brillouin-zone path Γ-K-M that is of primary interest for the comparison with experiment. In our MPS calculations we determine the time-dependent spin-spin correlation function

$$\begin{aligned} C^{\alpha\beta}_{\mathbf{r}}(\mathbf{x},t) &= \langle \hat{\mathcal{S}}^{\alpha}_{\mathbf{r+x}}(t)\hat{\mathcal{S}}^{\beta}_{\mathbf{r}}(0)\rangle \\ &= \langle [\hat{S}^{\alpha}_{\mathbf{r+x}}(t) - \langle \hat{S}^{\alpha}_{\mathbf{r+x}}(t)\rangle][\hat{S}^{\beta}_{\mathbf{r}}(0) - \langle \hat{S}^{\beta}_{\mathbf{r}}(0)\rangle]\rangle \\ &= \langle \hat{S}^{\alpha}_{\mathbf{r+x}}(t)\hat{S}^{\beta}_{\mathbf{r}}(0)\rangle - \langle \hat{S}^{\alpha}_{\mathbf{r+x}}(t)\rangle \, \langle \hat{S}^{\beta}_{\mathbf{r}}(0)\rangle \end{aligned} \tag{5}$$

where the disconnected part is subtracted in order to remove the Bragg peaks in C^{zz}. Using $|0\rangle$ to denote the ground state and E_0 for the ground-state energy of H, we rewrite Eq. (5) in the form

$$\begin{aligned} C^{\alpha\beta}_{\mathbf{r}}(\mathbf{x},t) = \langle 0|\hat{S}^{\alpha}_{\mathbf{r+x}}\, e^{-i(H-E_0)t}\, \hat{S}^{\beta}_{\mathbf{r}}|0\rangle \\ - \langle 0|\hat{S}^{\alpha}_{\mathbf{r+x}}|0\rangle\, \langle 0|\hat{S}^{\beta}_{\mathbf{r}}|0\rangle\,, \end{aligned} \tag{6}$$

where $\mathbf{r}$ is the site at which the initial spin operator is applied and $\mathbf{x}$ is the vector separation in the two-point correlator. Because of the residual U(1) symmetry of the Heisenberg Hamiltonian in a field, the total magnetization in the z direction is conserved under time evolution. We take advantage of this symmetry to increase the performance of the calculation by restricting the MPS to a single magnetization sector. It follows that only the correlators which satisfy $\alpha\beta \in \{zz, +-, -+\}$ are non-zero. The other spin-spin correlation functions can be obtained from the identity $C^{xx}_{\mathbf{r}}(\mathbf{x},t) \equiv C^{yy}_{\mathbf{r}}(\mathbf{x},t) \equiv \frac{1}{2}[C^{+-}_{\mathbf{r}}(\mathbf{x},t) + C^{-+}_{\mathbf{r}}(\mathbf{x},t)]$.

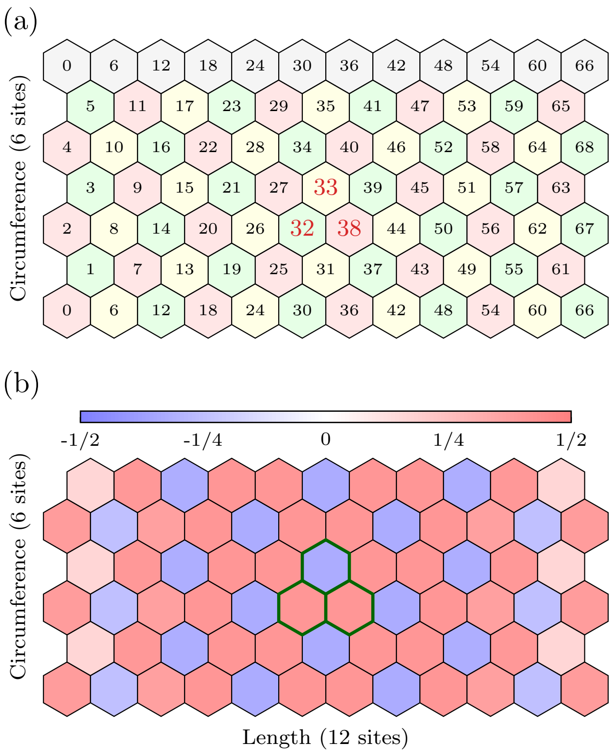

Supplementary Figure 13. (a) Example of the cylinder geometry used in our MPS calculations. The three sublattices are indicated by different colours. Numbers denote the index in the linear arrangement of the MPS and the three centre sites (used as initial excitation sites for the time evolution) are marked by red indices. Grey cells illustrate the wrapping specified by the periodic (XC) boundary conditions. (b) Local magnetization $\langle S^z_i \rangle$ measured in the ground state within the 1/3 magnetization plateau, showing up-up-down order. The three centre sites are marked by green boundaries.

In a typical time-evolution process, one selects a single site, $\mathbf{r}$, at the centre of the cylinder and uses a single time-evolved state to obtain the spectral function. However, the physics of the TLHAF in a field is such that there is a UUD-type symmetry-breaking of the z-axis magnetization at all finite fields. Our MPS set-up is therefore chosen such that the ground state in a field is a representative symmetry-broken state with a pronounced UUD-type local z-axis magnetization pattern, as shown in Supplementary Figure 13(b). In fact it is more efficient to repeat the dynamical evolution using three distinct starting sites than it is to use a single MPS with all three symmetry-broken states superposed in a spatially uniform way. The reason for this is that the approximate tripling of the bond dimension required to represent this symmetric MPS would lead to a much more expensive time evolution, because of the expected χ^3 scaling, than using three different starting states at a lower bond dimension does.

To obtain the spectral functions that correspond to the experiment, it is necessary to restore the lost translation symmetry. To this end, the spectral function $S_{\mathbf{r},\alpha\beta}(\mathbf{Q},\omega)$ in Eq. (3) of the main text is calculated for the three sites $\mathbf{r}$ corresponding to the three sublattices of the central unit cell of a sin-

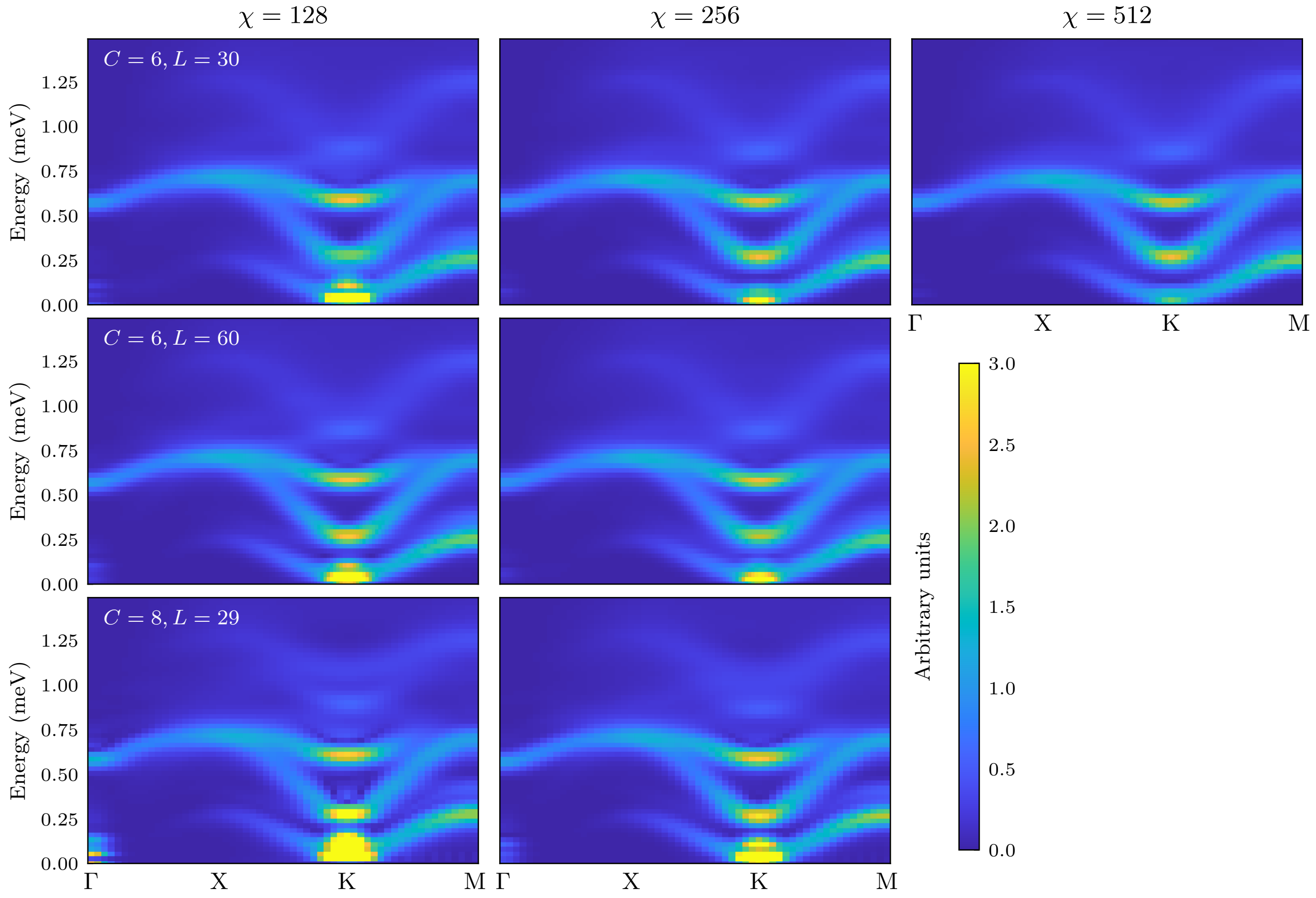

Supplementary Figure 14. Comparison of spectral functions computed using the MPS method for different system sizes and bond dimensions at the fixed values of the Gaussian envelope parameters stated in the text. Here we show the quantity $S_{xx}(\mathbf{Q},\omega) + S_{zz}(\mathbf{Q},\omega)$ at a magnetic field corresponding to the experimental value $B = 3$ T. The columns show increasing values of the bond dimension, χ, while the rows show cylinders of different sizes, as labelled in the panels in the first column.

gle symmetry-broken ground state (Supplementary Figure 13) and we take the equally weighted average to obtain the symmetric spectral function

$$S_{\alpha\beta}(\mathbf{Q},\omega) = \frac{1}{3}\sum_{\mathbf{r}} S_{\mathbf{r},\alpha\beta}(\mathbf{Q},\omega). \tag{7}$$

This procedure is repeated for all $\alpha\beta \in \{zz, +-, -+\}$, meaning that in total nine time-evolution runs are required at every value of the magnetic field, B.

In addition, symmetries of the Heisenberg Hamiltonian allow us to obtain the negative-time correlation functions at no additional computational cost as the complex conjugate of the positive-time ones, i.e. $C^{\alpha\beta}_{\mathbf{r}}(\mathbf{x},-t) = \overline{C^{\alpha\beta}_{\mathbf{r}}(\mathbf{x},t)}$. Putting these together, the symmetric spectral function of Eq. (7) simplifies to the form

$$S_{\alpha\beta}(\mathbf{Q},\omega) = \frac{2}{3}\sum_{\mathbf{r}}\int_0^\infty \mathrm{d}t \sum_{\mathbf{x}} e^{-i\,\mathbf{x}\cdot\mathbf{Q}} \left[\cos(\omega t)\,\mathrm{Re}\,C^{\alpha\beta}_{\mathbf{r}}(\mathbf{x},t) - \sin(\omega t)\,\mathrm{Im}\,C^{\alpha\beta}_{\mathbf{r}}(\mathbf{x},t)\right],$$

where the outer sum runs over the three centre sites. To compensate for the finite cylinder length and time-step series, it is standard before Fourier transformation to convolve the correlation function with a Gaussian envelope,

$$C^{\alpha\beta}_{\mathbf{r}}(\mathbf{x},t) \to e^{-\sigma_t t^2} e^{-\sigma_x(\mathbf{e}_L\cdot\mathbf{x})^2}\, C^{\alpha\beta}_{\mathbf{r}}(\mathbf{x},t), \tag{8}$$

which results in an effective broadening of the spectral function. For this we used $\sigma_t = 0.005 J_1^2$ and $\sigma_x = 0.02/a^2$, where a is the unit-cell size of the TL and $\mathbf{e}_L$ the unit vector along the cylinder axis, leading to the effective resolution in energy and momentum given in the Methods section of the main text.

In Supplementary Figure 14 we show spectra obtained using this procedure in the UUD (1/3-plateau) phase, at a magnetic field equivalent to $B = 3$ T, to illustrate the degree to which our calculations have converged to the spectral function of the infinite system. We observe that increasing the cylinder circumference from $C = 6$ to 8, the length from $L = 30$ to 60 or the bond dimension of the MPS from $\chi = 256$ to 512 make only minor changes to the quality of the spectra at the values of σ_t and σ_x chosen to match the experimental resolution. We comment that higher-resolution experimental data would require higher C, L or χ values to achieve convergence, and that our present MPS studies allow a factor-2 reduction in σ_t

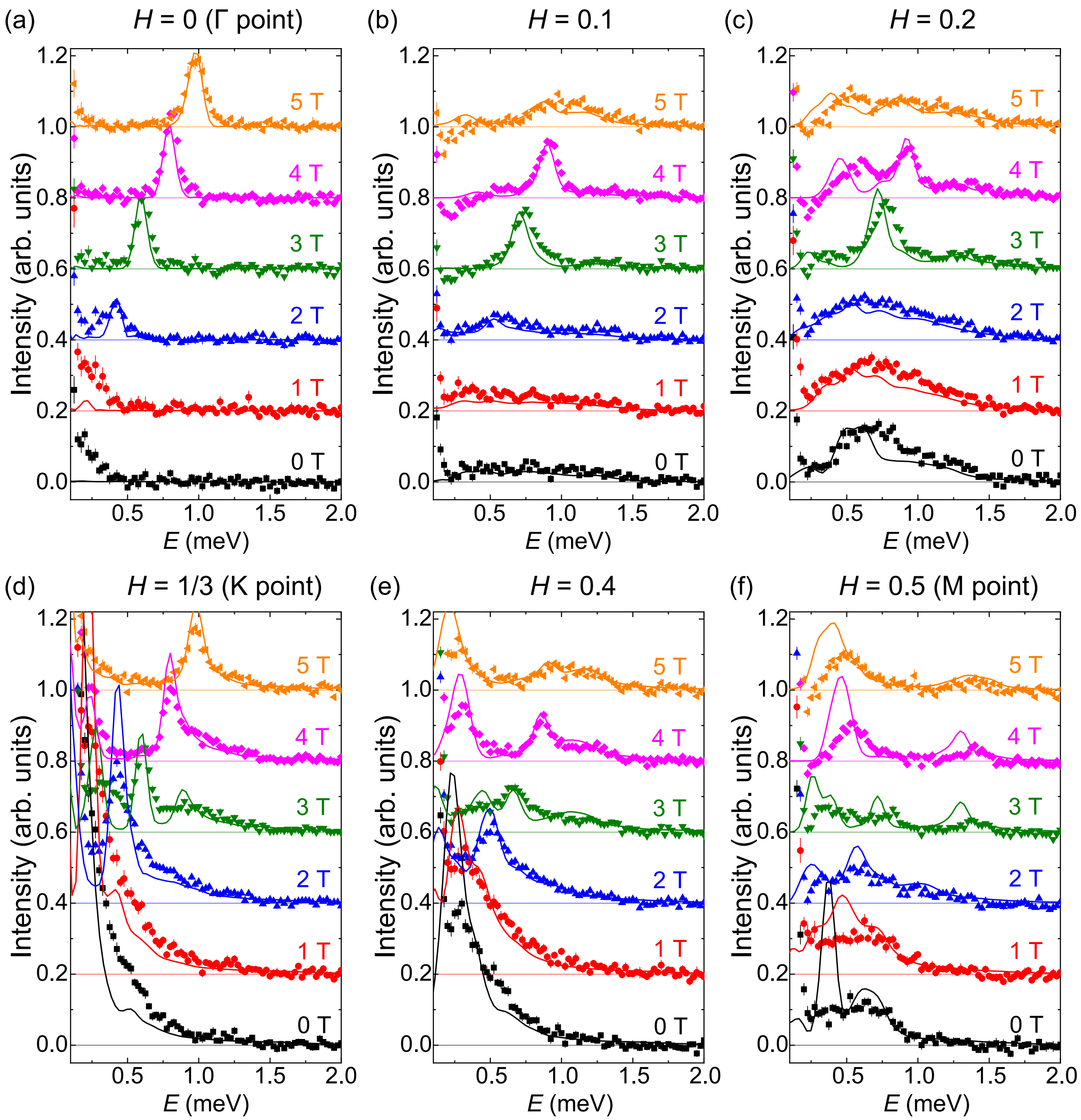

Supplementary Figure 15. Constant-$\mathbf{Q}$ cuts through the experimental (symbols) and calculated (solid lines) spectral functions for six different magnetic fields and six $\mathbf{Q}$ values along the $[H\ H\ 2.5]$ line. For clarity the cuts are shown with a vertical offset of 0.2.

and σ_x (meaning a factor of $\sqrt{2}$ better resolution) before artifacts appear at isolated $\mathbf{Q}$ values. All of the results shown in Figs. 1, 3 and 4 of the main text were obtained using $C = 6$ and $L = 30$, with χ set to 1024 for $B < 2$ T and to 512 for all higher fields. All correlation functions were evaluated up to a final time $t_{\max} = 90/J_1$, with a time step of $\Delta t = 0.1/J_1$.

Supplementary Note 6. COMPARISON OF MEASURED AND CALCULATED SPECTRAL FUNCTIONS

For a quantitative comparison of the MPS calculations with the experimental INS data, we present our results in two different ways. In Figs. 3b, 3d, 3f, 3h, 3j and 3l of the main text we show the spectral functions calculated for fields of 0, 2, 3, 4, 5 and 8 T with the symbols from Figs. 3a, 3c, 3e, 3g, 3i and 3k overlaid, which shows that the energies and wavevectors of all the characteristic spectral features are reproduced with quantitative accuracy. In Supplementary Figure 15 we show

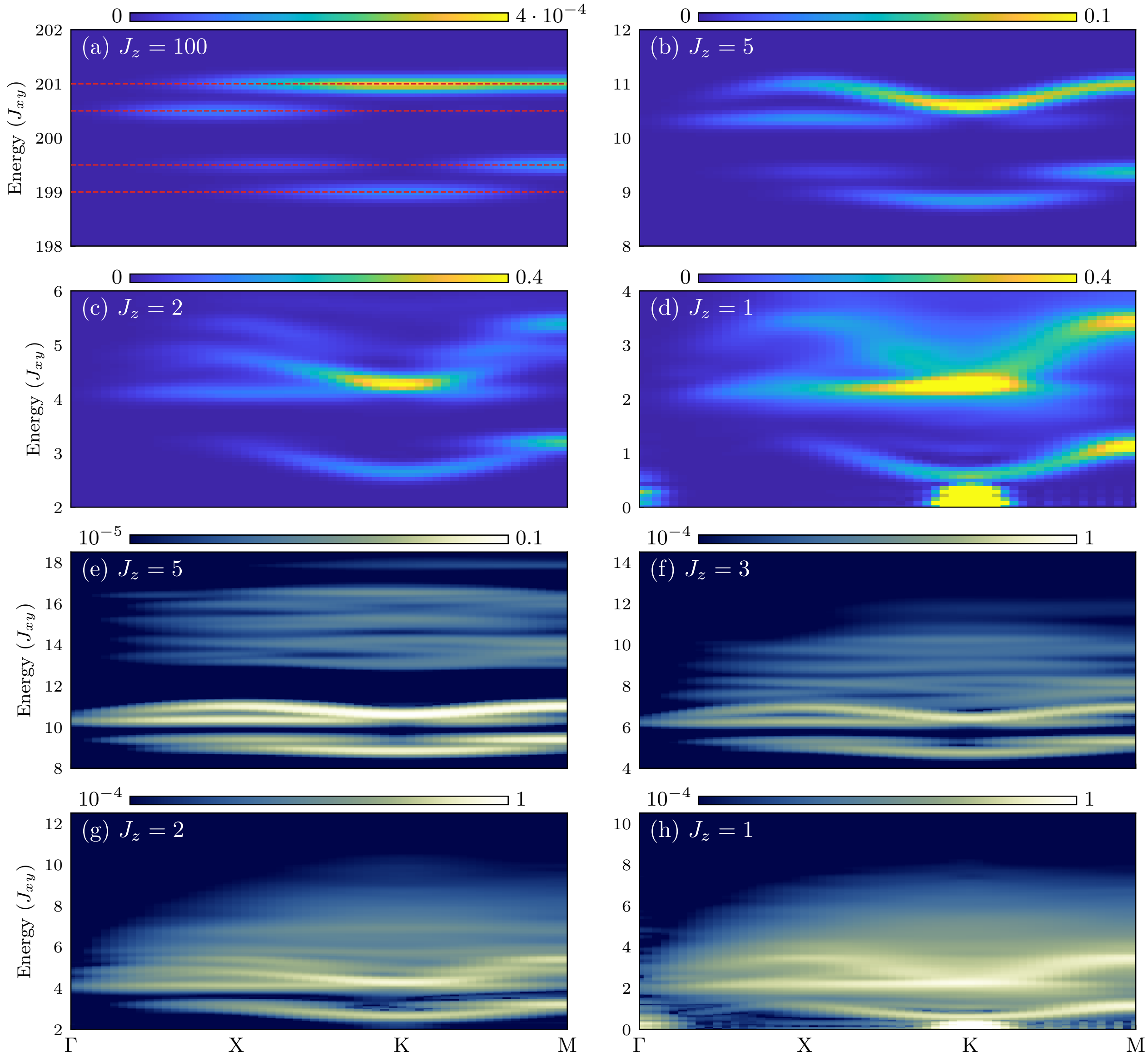

Supplementary Figure 16. Cylinder MPS calculations of the longitudinal spectral function for the nearest-neighbour $S = 1/2$ TLAF with XXZ spin interactions. (a-d) Two-magnon bound states obtained with anisotropies $J_z/J_{xy} = 100$ (a), 5 (b), 2 (c) and 1 (d). We draw attention to the energy scales on the y-axes: the centre of the bound-state spectrum is set by J_z and the width by J_{xy}. Dashed red lines in panel (a) show the four separate discrete levels obtained in the Ising limit. (e-h) Bound and scattering states shown on a logarithmic intensity scale for the anisotropy ratios $J_z/J_{xy} = 5$ (e), 3 (f), 2 (g) and 1 (h).

constant-**Q** cuts through the measured and calculated spectral functions for a range of **Q** points and applied magnetic fields. This extensive comparison reveals that the MPS calculations do an excellent job for all **Q** points at $B \geq 2$ T, losing their quantitative accuracy in locating the excitation features only on approaching the low-field limit ($B = 0$ and 1 T). Regarding the calculated intensities, we find that the MPS calculations achieve quantitative accuracy at low $|\mathbf{Q}|$ and between K and M, but clearly experience some challenges with the low-energy features near the K and M points.

Supplementary Note 7. BOUND STATES OF SPIN WAVES IN THE UUD PHASE

With the goal of understanding the excitation spectrum in the UUD phase of the $S = 1/2$ TLAF, we focus on excitations at the same total S^z as the 1/3-magnetization plateau. In order to elucidate the spectral response at the Heisenberg point, we consider an XXZ Hamiltonian with the magnetic field orientated parallel to the anisotropy axis,

$$\mathcal{H} = \sum_{\langle i,j \rangle} \tfrac{1}{2} J_{xy} \left(S_i^+ S_j^- + S_i^- S_j^+ \right) + J_z S_i^z S_j^z - h \sum_i S_i^z, \quad (9)$$

where $h = \mu_B g_{ab} B$. At $m = 1/3$, the ground state in the Ising limit, $J_z/J_{xy} \gg 1$, is the UUD product state illustrated in Fig. 4c of the main text. An excitation at the same S^z consists at least of one pair of local spin-flips, $U \rightarrow D$ and $D \rightarrow U$. If the two flipped spins are spatially well separated, they cost an energy $3J_z$ in the Ising limit, but if they are nearest neighbours then they cost only $2J_z$. We note that, because the total S^z is unchanged by two opposing spin flips, the z-axis magnetic field has no effect on the energies of these two-spin excitations.

As the next step we consider the perturbative limit with a finite, but small, J_{xy}. The nearest-neighbour bound states now show a spatially localized yet partially mobile structure. A schematic representation is shown in Fig. 4c of the main text, where the flipped red spin is confined, but can hop around the flipped blue spin along a finite chain of six sites, i.e. on the hexagon around the blue spin. The discrete energy spectrum of this six-site chain shows a lifting of the bound-state degeneracy to six flat bands with energies $\{2J_z + J_{xy}, 2J_z + J_{xy}/2, 2J_z - J_{xy}/2, 2J_z - J_{xy}\}$, with the second and third energies each twofold degenerate. The manifold of excitations arising from pairs of flipped spins far away from each other is also split at first order in J_{xy}, and forms a two-particle continuum at an energy around $3J$ whose support can be derived from the dispersion of the single spin-flips.

The next step is to determine whether this structure of bound states is visible in the longitudinal dynamical structure factor, $S_{zz}(\mathbf{Q}, \omega)$. For this we performed MPS calculations for the nearest-neighbour model at different values of J_z/J_{xy}, and in Supplementary Figure 16(a) we show the spectral function at $J_z/J_{xy} = 100$ along the usual Γ-K-M path in the Brillouin zone. Indeed one observes that the spectral weight becomes $\mathbf{Q}$-dependent, and that the excitation energies are confined to the perturbative branches (horizontal dashed red lines) discussed above. As J_z/J_{xy} is lowered to 5 [Supplementary Figure 16(b)], 2 [Supplementary Figure 16(c)] and the Heisenberg point [Supplementary Figure 16(d)], the bound-state branches acquire an increasing dispersion and broaden in energy; in particular, while the lower bound state remains relatively sharp as $J_z/J_{xy} \rightarrow 1$, the upper scattering states broaden into the bow-tie structure of continuum IV.

To track the origin of this broadening, in Supplementary Figure 16(e-h) we show four spectral functions on a logarithmic intensity scale. In Supplementary Figure 16(e), which corresponds to Supplementary Figure 16(b), the two-magnon scattering continuum begins to become visible, centred at an energy around $3J_z$ ($15J_{xy}$). As J_z/J_{xy} is lowered, this continuum rises in intensity and overlaps increasingly with the energy window of the bound states, until at the Heisenberg point [Supplementary Figure 16(h)] only a part of the lowest bound-state branch remains as the isolated and well defined mode observed in experiment (Fig. 4a of the main text).

[1] J. Rodríguez-Carvajal, Recent advances in magnetic structure determination by neutron powder diffraction, Physica B: Condens. Matter **192**, 55 (1993).

[2] J. Xing, L. D. Sanjeewa, J. Kim, G. R. Stewart, M.-H. Du, F. A. Reboredo, R. Custelcean, and A. S. Sefat, CSD 1952075: Experimental Crystal Structure Determination 10.25505/fiz.icsd.cc23j954 (2020).

[3] A. O. Scheie, E. A. Ghioldi, J. Xing, J. A. M. Paddison, N. E. Sherman, M. Dupont, D. Abernathy, D. M. Pajerowski, S.-S. Zhang, L. O. Manuel, A. E. Trumper, C. D. Pemmaraju, A. S. Sefat, D. S. Parker, T. P. Devereaux, J. E. Moore, C. D. Batista, and D. A. Tennant, Witnessing quantum criticality and entanglement in the triangular antiferromagnet $KYbSe_2$, arXiv:2109.11527.

[4] P.-L. Dai, G. Zhang, Y. Xie, C. Duan, Y. Gao, Z. Zhu, E. Feng, Z. Tao, C.-L. Huang, H. Cao, A. Podlesnyak, G. E. Granroth, M. S. Everett, J. C. Neuefeind, D. Voneshen, S. Wang, G. Tan, E. Morosan, X. Wang, H.-Q. Lin, L. Shu, G. Chen, Y. Guo, X. Lu, and P. Dai, Spinon Fermi Surface Spin Liquid in a Triangular Lattice Antiferromagnet $\mathrm{NaYbSe_2}$, Phys. Rev. X **11**, 021044 (2021).

[5] R. Kaneko, S. Morita, and M. Imada, Gapless Spin-Liquid Phase in an Extended Spin 1/2 Triangular Heisenberg Model, J. Phys. Soc. Jpn. **83**, 093707 (2014).

[6] P. H. Y. Li, R. F. Bishop, and C. E. Campbell, Quasiclassical magnetic order and its loss in a spin-$\frac{1}{2}$ Heisenberg antiferromagnet on a triangular lattice with competing bonds, Phys. Rev. B **91**, 014426 (2015).

[7] Z. Zhu and S. R. White, Spin liquid phase of the $S = \frac{1}{2}$ J_1-J_2 Heisenberg model on the triangular lattice, Phys. Rev. B **92**, 041105 (2015).

[8] W.-J. Hu, S.-S. Gong, W. Zhu, and D. N. Sheng, Competing spin-liquid states in the spin-$\frac{1}{2}$ Heisenberg model on the triangular lattice, Phys. Rev. B **92**, 140403 (2015).

[9] Y. Iqbal, W.-J. Hu, R. Thomale, D. Poilblanc, and F. Becca, Spin liquid nature in the Heisenberg $J_1 - J_2$ triangular antiferromagnet, Phys. Rev. B **93**, 144411 (2016).

[10] S. Hu, S. Eggert, and Y.-C. He, Dirac Spin Liquid on the Spin-1/2 Triangular Heisenberg Antiferromagnet, Phys. Rev. Lett. **123**, 207203 (2019).

[11] J. Xing, L. D. Sanjeewa, A. F. May, and A. S. Sefat, Synthesis and anisotropic magnetism in quantum spin liquid candidates $AYbSe_2$ (A= K and Rb), APL Materials **9**, 111104 (2021).

[12] K. M. Ranjith, D. Dmytriieva, S. Khim, J. Sichelschmidt, S. Luther, D. Ehlers, H. Yasuoka, J. Wosnitza, A. A. Tsirlin, H. Kühne, and M. Baenitz, Field-induced instability of the quantum spin liquid ground state in the $J_{\mathrm{eff}} = \frac{1}{2}$ triangular-lattice compound $\mathrm{NaYbO_2}$, Phys. Rev. B **99**, 180401 (2019).

[13] K. M. Ranjith, S. Luther, T. Reimann, B. Schmidt, P. Schlender, J. Sichelschmidt, H. Yasuoka, A. M. Strydom, Y. Skourski, J. Wosnitza, H. Kühne, T. Doert, and M. Baenitz, Anisotropic field-induced ordering in the triangular-lattice quantum spin liquid $\mathrm{NaYbSe_2}$, Phys. Rev. B **100**, 224417 (2019).

[14] L. Ding, P. Manuel, S. Bachus, F. Grußler, P. Gegenwart, J. Singleton, R. D. Johnson, H. C. Walker, D. T. Adroja, A. D. Hillier, and A. A. Tsirlin, Gapless spin-liquid state in the structurally disorder-free triangular antiferromagnet $\mathrm{NaYbO_2}$, Phys. Rev. B **100**, 144432 (2019).

[15] O. Young, A. R. Wildes, P. Manuel, B. Ouladdiaf, D. D. Khalyavin, G. Balakrishnan, and O. A. Petrenko, Highly frustrated magnetism in $\mathrm{srho_2o_4}$: Coexistence of two types of short-range order, Phys. Rev. B **88**, 024411 (2013).

[16] J. Xing, L. D. Sanjeewa, J. Kim, G. R. Stewart, A. Podlesnyak, and A. S. Sefat, Field-induced magnetic transition and spin fluctuations in the quantum spin-liquid candidate $\mathrm{CsYbSe_2}$, Phys. Rev. B **100**, 220407 (2019).

[17] S. Toth and B. Lake, Linear spin wave theory for single-Q incommensurate magnetic structures, J. Phys. Condens. Matter **27**, 166002 (2015).

[18] A. V. Chubokov and D. I. Golosov, Quantum theory of an antiferromagnet on a triangular lattice in a magnetic field, J. Phys. Condens. Matter **3**, 69 (1991).

[19] C. Hotta, S. Nishimoto, and N. Shibata, Grand canonical finite size numerical approaches in one and two dimensions: Real space energy renormalization and edge state generation, Phys. Rev. B **87**, 115128 (2013).

[20] J. Hauschild and F. Pollmann, Efficient numerical simulations with Tensor Networks: Tensor Network Python (TeNPy), SciPost Phys. Lect. Notes **5**, 1 (2018).

[21] A. Szasz, J. Motruk, M. P. Zaletel, and J. E. Moore, Chiral Spin Liquid Phase of the Triangular Lattice Hubbard Model: A Density Matrix Renormalization Group Study, Phys. Rev. X **10**, 021042 (2020).
